# Early detection and improved genomic surveillance of SARS-CoV-2 variants from deep sequencing data

**DOI:** 10.1016/j.isci.2022.104487

**Published:** 2022-05-30

**Authors:** Daniele Ramazzotti, Davide Maspero, Fabrizio Angaroni, Silvia Spinelli, Marco Antoniotti, Rocco Piazza, Alex Graudenzi

**Affiliations:** 1 Department of Medicine and Surgery, University of Milan-Bicocca, Monza, Italy; 2 Department of Informatics, Systems and Communication, University of Milan-Bicocca, Milan, Italy; 3 Institute of Molecular Bioimaging and Physiology, National Research Council (IBFM-CNR), Segrate, Milan, Italy; 4 CNAG-CRG, Centre for Genomic Regulation (CRG), Barcelona Institute of Science and Technology (BIST), Barcelona, Spain; 5 Bicocca Bioinformatics, Biostatistics and Bioimaging Centre – B4, Milan, Italy

**Keywords:** microbiology, virology, bioinformatics, genomic analysis

## Abstract

A key task of genomic surveillance of infectious viral diseases lies in the early detection of dangerous variants. Unexpected help to this end is provided by the analysis of deep sequencing data of viral samples, which are typically discarded after creating consensus sequences. Such analysis allows one to detect intra-host low-frequency mutations, which are a footprint of mutational processes underlying the origination of new variants. Their timely identification may improve public-health decision-making with respect to traditional approaches exploiting consensus sequences. We present the analysis of 220,788 high-quality deep sequencing SARS-CoV-2 samples, showing that many spike and nucleocapsid mutations of interest associated to the most circulating variants, including Beta, Delta, and Omicron, might have been intercepted several months in advance. Furthermore, we show that a refined genomic surveillance system leveraging deep sequencing data might allow one to pinpoint emerging mutation patterns, providing an automated data-driven support to virologists and epidemiologists.

## Highlights

• Early detection of hazardous variants is crucial in genomic surveillance of epidemics
• Most approaches focus on consensus sequences and neglect intra-host minor mutations
• We present the analysis of minor mutation profiles from 220k + SARS-CoV-2 samples
• Many S and N mutations of interest are detected as minor several months in advance

## Graphical abstract

**Figure:**
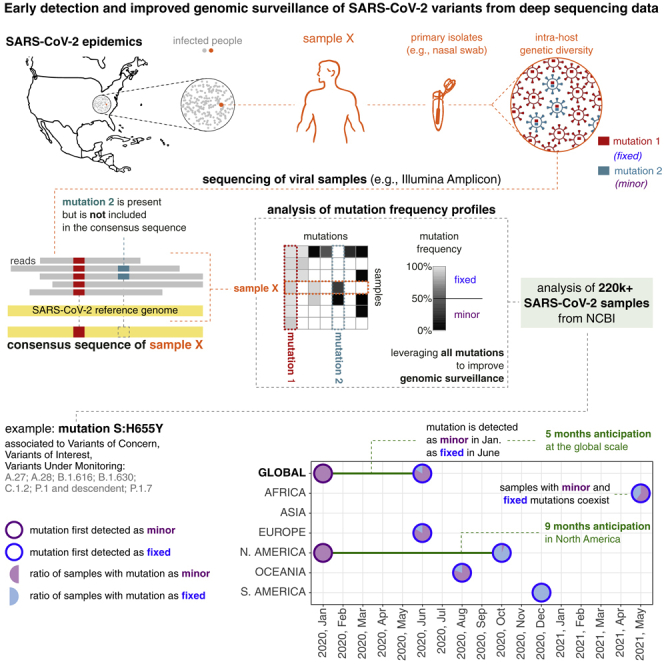

## Introduction

The dramatic impact of the COVID-19 pandemic at the global scale has proven that pivotal efforts must be devoted by the scientific community to the timely identification and quantification of hazardous variants, i.e., those showing increased virulence, pathogenesis, or ability to escape therapeutic strategies such as vaccines (Oude Munnink et al., 2021; Elliott et al., 2021). To this end, institutions such as the World Health Organization (WHO), the European Centre for Disease Prevention and Control (ECDC), the Centers for Disease Control and Prevention (CDC), and others are repeatedly updating the lists of the so-called variants of interests (VOIs), variants of concern (VOCs), variants under monitoring (VUMs) (also named Variants Being Monitored [VBMs]) and de-escalated aminos (DEVs) (World Health Organization, 2021; European Centre for Disease Prevention and Control, 2021; Centers for Disease Control and Prevention, 2021).

Each variant is identified and categorized according to institution-specific molecular and epidemiological criteria (Konings et al., 2021). VOCs are usually associated with evidence of diminished effectiveness of treatments, increased transmissibility, immune escape, and/or diagnostic escape. VOIs present genetic changes that are predicted or known to cause the same effect of a VOC but have a limited prevalence (e.g., in circumscribed outbreak clusters). VUMs bear genetic markers suspected to impact the epidemic dynamics but circulate at a very low level. Finally, a variant is classified as DEV if it is no longer circulating or if there is solid evidence that it does not affect the overall epidemiological situation.

From the molecular perspective, each variant is associated to one or more (sub)lineages of the SARS-CoV-2 phylogenetic model (in this work, we adopt the Pango lineage nomenclature [Rambaut et al., 2020]) and is characterized by sets of distinct alterations of the viral genome, i.e., single-nucleotide and indels, including the so-called Spike Mutations of Interest (SMoIs) (Hodcroft, 2021). Single-nucleotide alterations are typically labeled with the syntax: [*ORF_name*][:][*reference_amino_acid*][*ORF_codon*][*mutated_amino_acid*], whereas deletions with: [*ORF_name*][:][*reference_amino_acid*][*ORF_codon*][-]. For instance, the SMoI S:D614G indicates the single-nucleotide alteration(s) leading to the synthesis of the G amino acid (Glycine) instead of the D amino acid (Aspartic Acid) in the 614^th^ codon of the Spike (S) ORF of the SARS-CoV-2 genome. In Table 1, one can find the set of SARS-CoV-2 variants listed as VOI, VOC, VUM, or DEV by at least one of the three public health bodies as of October 26th 2021, and the related WHO label (in Greek letters), Pango lineage, and set of SMoIs (note that the Omicron variant was added to the list, despite being designed as VOC by the WHO on November 26th 2021). For further details on the categorization criteria please refer to the institution websites, whereas for an up-to-date association between mutations, variants, and lineages please refer to Hodcroft (2021).

**Table 1:** Hazardous SARS-CoV-2 variants

WHO label	Pango lineage	Spike mutations of interest (SMoI)	ECDC category	CDC category	WHO category	Early detection	Refined surveillance
Alpha	B.1.1.7 and Q lineages	S:N501Y, S:D614G, S:P681H	DEV	VUM	VOC		✓
Alpha+	B.1.1.7 + S:L452R	S:L452R, S:N501Y, S:D614G, S:P681H	DEV	VUM	VOC	✓	
Alpha+	B.1.1.7 + S:E484K	S:E484K, S:N501Y, S:D614G, S:P681H	DEV	VUM	VOC		✓
Alpha+	B.1.1.7 + S:S494P	S:S494P, S:N501Y, S:D614G, S:P681H	DEV	VUM	VOC		✓
Beta	B.1.351 and descendent	S:K417N, S:E484K, S:N501Y, S:D614G, S:A701V	VOC	VUM	VOC	✓	✓
Beta+	B.1.351 + S:L18F	S:L18F, S:K417N, S:E484K, S:N501Y, S:D614G, S:A701V	VOI	VUM	VOC	✓	✓
Beta+	B.1.351 + S:P384L	S:P384L, S:K417N, S:E484K, S:N501Y, S:D614G, S:A701V	VOI	VUM	VOC	✓	✓
Beta+	B.1.351 + S:E516Q	S:K417N, S:E484K, S:N501Y, S:E516Q, S:D614G, S:A701V	VOI	VUM	VOC	✓	
Gamma	P.1 and descendent	S:K417T, S:E484K, S:N501Y, S:D614G, S:H655Y	VOC	VUM	VOC	✓	
Gamma+	P.1.7	S:K417T, S:E484K, S:N501Y, S:D614G, S:H655Y, S:P681H	VOI	VUM	VOC	✓	✓
Delta	B.1.617.2	S:L452R, S:T478K, S:D614G, S:P681R	VOC	VOC	VOC	✓	✓
Delta+	AY lineages	S:K417N, S:L452R, S:T478K, S:D614G, S:P681R	VOI	VOC	VOC	✓	
Delta+	AY.34	S:L452R, S:T478K, S:D614G, S:Q677H, S:P681R	VOI	VOC	VOC	✓	✓
Delta+	B.1.617.2 + S:E484X	S:L452R, S:T478K, S:E484X, S:D614G, S:P681R	VOI	VOC	VOC	✓	
Delta+	B.1.617.2 + S:Q613H	S:L452R, S:T478K, S:Q613H, S:D614G, S:P681R	VOI	VOC	VOC	✓	✓
Epsilon	B.1.427 and B.1.429	S:L452R, S:D614G	DEV	VUM	VUM	✓	
Zeta	P.2	S:E484K, S:D614G	DEV	VUM	–		
Eta	B.1.525	S:E484K, S:D614G, S:Q677H	DEV	VUM	VUM		✓
Theta	P.3	S:E484K, S:N501Y, S:D614G, S:P681H	DEV	–	–		
Iota	B.1.526	S:E484K, S:D614G, S:A701V	DEV	VUM	VUM	✓	
Kappa	B.1.617.1	S:L452R, S:E484Q, S:D614G, S:P681R	DEV	VUM	VUM	✓	✓
Lambda	C.37	S:L452Q, S:F490S, S:D614G	VOI	–	VOI		✓
Mu	B.1.621 and B.1.621.1	S:R346K, S:E484K, S:N501Y, S:D614G, S:P681H	VOI	VUM	VOI		✓
Omicron^a^	B.1.1.529	S:K417N, S:S477N, S:T478K, S:N501Y, S:D614G, S:H655Y, S:N679K, S:P681H	VOC	VOC	VOC	✓	
–	A.23.1	S:V367F, S:E484K, S:Q613H	DEV	–	–		✓
–	A.27	S:L452R, S:N501Y, S:A653V, S:H655Y	DEV	–	–	✓	
–	A.28	S:E484K, S:N501T, S:H655Y	DEV	–	–	✓	
–	AY.4.2	S:Y145H, S:A222V, S:L452R, S:T478K, S:D614G, S:P681R	VUM	–	–	✓	
–	AT.1	S:E484K, S:D614G, S:N679K	DEV	–	–		
–	AV.1	S:N439K, S:E484K, S:D614G, S:P681H	DEV	–	–		
–	B.1.1.318	S:E484K, S:D614G, S:P681H	VUM	–	VUM		✓
–	B.1.1.519	S:T478K, S:D614G	DEV	–	VUM	✓	✓
–	B.1.1.523	S:E484K, S:S494P, S:D614G	–	–	VUM		
–	B.1.214.2	S:Q414K, S:N450K, S:D614G	DEV	–	VUM	✓	✓
–	B.1.466.2	S:N439K, S:D614G, S:P681R	–	–	VUM		
–	B.1.616	S:V483A, S:D614G, S:H655Y, S:G669S	DEV	–	–	✓	
–	B.1.617.3	S:L452R, S:E484Q, S:D614G, S:P681R	DEV	VUM	–	✓	✓
–	B.1.619	S:E484K, S:D614G	–	–	VUM		
–	B.1.620	S:S477N, S:E484K, S:D614G, S:P681H	DEV	–	VUM		
–	B.1.630	S:A222V, S:L452R, S:E484Q, S:D614G, S:H655Y	–	–	VUM	✓	
–	C.1.2	S:D614G, S:E484K, S:H655Y, S:N501Y, S:N679K, S:Y449H	VUM	–	VUM	✓	
–	C.16	S:L452R, S:D614G	DEV	–	–	✓	
–	C.36.3	S:L452R, S:D614G, S:Q677H	VUM	–	VUM	✓	
–	R.1	S:E484K, S:D614G	–	–	VUM		

List of SARS-CoV-2 variants of concern (VOC), of interest (VOI), under monitoring (VUM), and de-escalated variants (DEV), updated on October 26th, 2021, as from the categorization of World Health Organization (2021); European Centre for Disease Prevention and Control (2021); Centers for Disease Control and Prevention (2021). Omicron variant was added to the list even if designated as VOC on November 26th. Information on the WHO label, the constituting Pango lineages Rambaut et al. (2020), the associated spike mutations of interest (SMoI), and the institution-specific categories are shown. Variant labels marked with “+” include additional SMoIs with respect to the related upstream variant. In the last two columns, we report the variants for which either an early detection of the related SMoIs and/or a refined surveillance (via homoplasy analysis) is granted by exploiting deep sequencing data (see Results). Notice that A.23.1 and B.1.525 (Eta) are included in the list of so-called variants of note in the Cov-Lineages.org lineage report O’Toole et al. (2021a, 2021b).

a Notice that, at the time of writing, no SMoIs were explicitly associated to the Omicron variant. Here, we indicate the S mutations present in such variant identified as SMoI in at least one of the remaining 43 variants included in the table, whereas for an updated characterization of Omicron and other variants we refer the reader to Hodcroft (2021).

**Figure 1:**
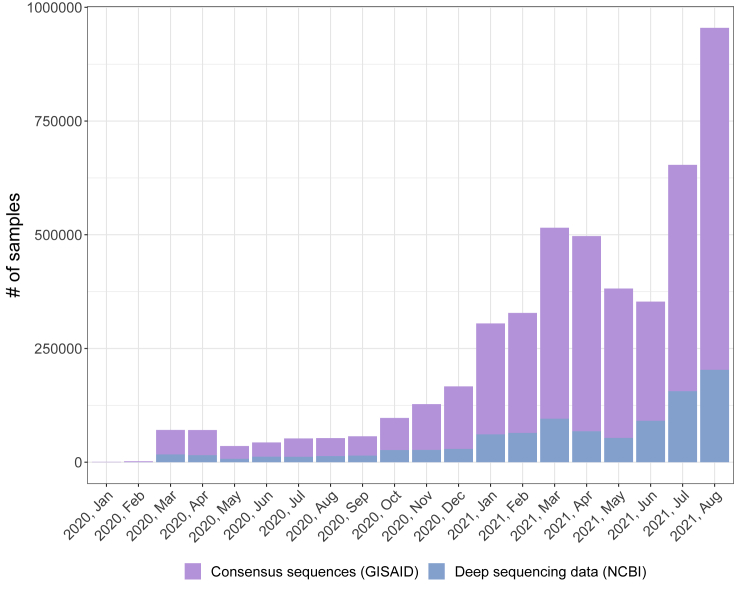
SARS-CoV-2 samples in GISAID and NCBI public repositories Number of SARS-CoV-2 samples for which either deep sequencing data or consensus sequences are available, grouped by month in which the related dataset is released in the period January 2020–August 2021. Source databases are NCBI (National Center for Biotechnology Information, 2021) for deep sequencing data and GISAID (Shu and McCauley, 2017) for consensus sequences (update: August 2021).

During the pandemic, the analysis and surveillance of SARS-CoV-2 variants has benefited from the surge of Next-Generation Sequencing (NGS) experiments performed via different protocols (e.g., Illumina RNA-seq or Amplicon) on viral samples, which are typically collected from primary isolates of infected people. The data generated are made available on portals such as GISAID (Shu and McCauley, 2017), Nextstrain (Hadfield et al., 2018), Cov-Lineages.org (O’Toole et al., 2021a, 2021b), NCBI SARS-CoV-2 Resources (National Center for Biotechnology Information, 2021), or the EMBL-EBI COVID-19 Data Portal (EMBL-EBI Covid-19 Data Portal, 2021). However, the large majority of available datasets include only the *consensus sequences* of the samples, rather than the source deep sequencing data from which such sequences are generated with distinct criteria. In fact, the proportion of deep sequencing datasets shared on public repositories has been significantly lower than that of consensus sequence during the course of the pandemic. To provide a clarifying example, we focus on two of the most widely used portals collecting NGS data of viral samples: as of August 2021, 4,558,675 consensus sequences are stored on the GISAID database (Shu and McCauley, 2017), whereas only 975,767 samples (≈21.4%) are included in (Illumina paired-end) sequencing datasets accessible (from different sources) on the NCBI website (National Center for Biotechnology Information, 2021), of which only approximately 50% are of high quality (see Figure 1 and see STAR Methods for further details). Furthermore, to the best of our knowledge, all existing portals collecting deep sequencing datasets do not currently provide any unified standard for their processing and analysis, nor any automated computational tool for their integration and in-depth investigation. Hence, the retrieval, processing, and analysis of such datasets may require a significant amount of ad hoc manual work and computation time, possibly hindering their proper exploitation.

Deep sequencing data are indeed essential to characterize the intra-host mutational landscape of viral samples and, especially, to identify the presence of minor mutations, i.e., single-nucleotide variants (SNVs) and indels detected with *low mutation frequency* (MF, which—roughly—is the ratio of alternative allele reads over the total reads for a specific genome position) (Liu et al., 2022). Minor mutations are—by definition—not included in consensus sequences but characterize the heterogeneous ensemble of viral subpopulations, known as *quasispecies* (Domingo et al., 2012; Knyazev et al., 2021), which are typically present in single hosts. Most important, every new genomic mutation first originates as minor within single hosts, due to replication errors of viral polymerases that are often induced by host-related mutational processes (Graudenzi et al., 2021; Maspero et al., 2021; Ramazzotti et al., 2022).

*Minor mutations* are generally purified, due to detrimental effects on the fitness of the virus, and are significantly affected by transmission events (e.g., bottlenecks), which hamper their diffusion in the population (see Gallego-García et al., 2022), as opposed to *fixed mutation*, which are usually transmitted from a host to another during infections. However, certain minor mutations may provide the virus with a fitness advantage, for instance in terms of enhanced reproductive potential or increased infectiveness and, accordingly, are positively selected, first within hosts and, later, in the population of infected people. For these and many additional transmission-related phenomena, the MF of certain minor mutations can sometimes increase both within single hosts and across infection chains. Once the MF exceeds a certain threshold (typically around ∼50%), the mutation fixates, meaning that the related viral subpopulation has become dominant within a given host. Only then the mutation is included into consensus sequences. Among the set of fixed mutations, some (e.g., the SMoIs) will eventually contribute to the origination of hazardous variants, subsequently impacting the course of the epidemic.

A finer characterization of the minor mutation landscape is therefore beneficial to (1) intercept impactful mutations prior to their fixation and (2) assess the presence of dangerous minor mutations in samples exhibiting circulating variants. Both aspects are essential for the definition of an effective genomics-informed epidemiological surveillance system, which may drive the design of timely public-health interventions, with substantial repercussions in terms of epidemiological dynamics and socioeconomic costs (Gardy and Loman, 2018; Baud et al., 2020; Verschuur et al., 2021; Davis et al., 2021). In this regard, note that the characterization of the minor mutation landscape has already been successfully exploited to investigate drug resistance, contagion chains, bottleneck effects, and mutational signatures, for a number of infectious diseases including COVID-19 (Beerenwinkel and Zagordi, 2011; Knyazev et al., 2021; Ramazzotti et al., 2021; Graudenzi et al., 2021).

In support of our claims, here we provide the largest up-to-date worldwide study of deep sequencing data of SARS-CoV-2 samples, which includes 220,788 high-quality samples from 137 distinct datasets. Our analyses are first focused on a list of 44 variants and related 35 SMoIs, included in the lists of hazardous variants by the WHO, the ECDC, and the CDC. We also analyze a list of 95 further spike mutations that have not been associated to any known variant at the time of writing but display significant diffusion patterns. Because attention was recently raised on the functional role of mutations hitting the nucleocapsid (N) protein (via the analysis of SARS-CoV-2 virus-like particles [Syed et al., 2021]), we also investigated a list of 13 N mutations with potential functional effect (here labelled as *N mutations of interest*, NMoIs) and a further list of 82 highly diffused N mutations.

In brief, (1) we prove that the identification of several S and N mutations could be anticipated of a significant time-span with respect to standard analyses based on consensus sequences, and (2) we highlight that a significant number of samples harboring circulating variants display homoplastic minor mutations, which might lead to the origination of new variants.

## Results

The analyses presented in this work focus on (1) the set of 35 S mutations (SMoIs) associated to the 44 SARS-CoV-2 variants included in the lists of VOCs, VOIs, VUMs, or DEVs in at least one of the WHO, the CDC, and the ECDC websites, as of October 26th 2021 (see Table 1; Omicron variant was labeled as VOC on November 26th 2021); (2) a list of additional 95 S mutations significantly diffused in the population; (3) a list of 13 N mutations with potential functional effect identified in (Syed et al., 2021) (NMoIs); and (4) a further list of highly diffused 82 N mutations. No considerations on the molecular properties or the epidemiological features of such mutations/variants are purposely reported, for which the readers are referred to the related literature.

### Early detection of mutations of interest

#### Spike mutations of interest associated to known variants

One of the major differences in employing deep sequencing data instead of consensus sequences lies in the possibility of detecting, in principle, any genomic mutation with great temporal advance. To corroborate this claim, we first analyzed separately each SMoI associated to the hazardous variants included in Table 1. A total amount of 35 distinct mutations were analyzed, involved in 44 variants (we recall that any variant can be associated to one or more Pango lineages [Rambaut et al., 2020]).

In particular, we computed (1) the MF of all SMoIs in all samples, (2) the prevalence of each SMoI in the population (i.e., proportion of samples) when detected as either minor (MF ≥5% and <50%) or fixed (MF ≥50%), with respect to collection date (grouped by month) and location (grouped by continent) (Note that we restricted the detection time analysis on the months in which any mutation is detected [with MF ≥5%] in at least 5 samples in a given location, so to ensure a minimum level of statistical significance [for a discussion on filtering criteria, please refer to the Limitations of the study section]).

**Figure 2:**
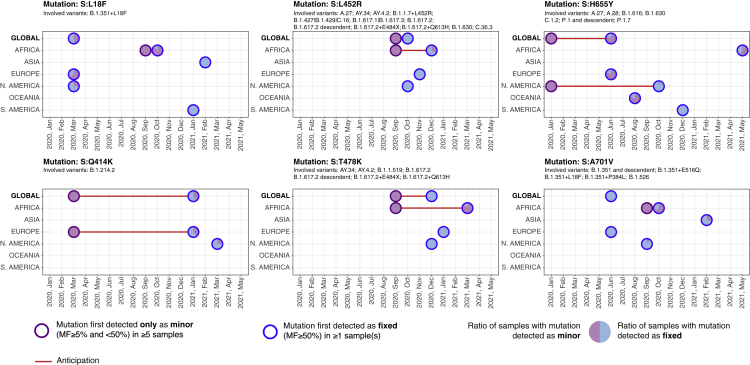
Early detection of 6 SMoIs associated to hazardous variants from deep sequencing data Analysis of SMoIs: S:L18F, S:Q414K, S:L452R, S:T478K, S:H655Y, and S:A701V (see Table 1). Circles with *purple* borders mark the first month in which the mutation was detected as minor (MF ≥5% and <50%) in at least 5 samples, while been still undetected as fixed (MF ≥50%); circles with *blue* borders mark the month in which the mutation was first detected as fixed in at least 1 sample; red lines highlight the anticipation (when >1 months). The analysis is performed by splitting the samples in the 6 distinct geographical regions and by reporting the corresponding results at the global scale. All circles contain a pie-chart that displays the ratio of samples showing that mutation either as minor or as fixed in that month (further details are provided in the main text). For each SMoI the related variants are also reported.

Overall, 4 (out of 35) SMoIs were detected as minor (in at least 5 samples) at least one month prior to their initial detection as fixed at the global level, i.e., S:Q414K in March 2020, S:L452R in September 2020, and S:H655Y in January 2020, and 2 additional SMoIs in at least one of the considered geographical regions, i.e., S:L18F, S:T478K, and S:A701V, in September 2020 in Africa (see Figure 2). The 6 SMoIs detected in advance characterize a large number of hazardous variants (see Table 1) and, in particular, S:Q414K is associated to one variant (B.1.214.2); S:L452R to 14 variants (B.1.1.7 + L452R (Alpha+), B.1.617.2 (Delta), AY lineages (Delta+), AY.34 (Delta+), B.1.617.2 + E484X (Delta+), B.1.617.2 + Q613H (Delta+), B.1.427 and B.1.429 (Epsilon), B.1.617.1 (Kappa), A.27, AY.4.2, B.1.617.3, B.1.630, C.16, C.36.3); S:H655Y to 8 variants (P.1 and descendent (Gamma), P.1.7 (Gamma+), B.1.1.529 (Omicron), A.27, A.28, B.1.616, B.1.630, C.1.2); S:L18F to one variant (B.1.351 + L18F (Beta+)); S:T478K to 8 variants (B.1.617.2 (Delta), AY lineages (Delta+), AY.34 (Delta+), B.1.617.2 + E484X (Delta+), B.1.617.2 + Q613H (Delta+), B.1.1.529 (Omicron), AY.4.2, B.1.1.519); and S:A701V to 5 variants (B.1.351 and descendent (Beta), B.1.351 + L18F (Beta+), B.1.351 + P384L (Beta+), B.1.351 + E516Q (Beta+), B.1.526 (Iota)).

Moreover, 4 additional SMoIs were initially detected *both* as minor (in at least 5 samples) and fixed (in at least 1 sample) at the global scale (S:V367F, S:K417N, S:A653V, S:Q677H) and another SMoI in at least one geographical region (S:D614G in North America), demonstrating that the circulation of SMoIs can be underestimated by considering consensus sequences only.

In Figure 3, one can find the in-depth analysis of mutations S:L452R and S:H655Y, whereas the analysis of all remaining early detected S and N mutations is presented in Figures S3–S36. More in detail, at the global scale, mutation S:L452R is first observed as minor in Africa in September 2020 and as fixed in North America in October 2020 (1 month in advance). The anticipation is remarkably amplified when considering the local scale. In fact, in Africa the mutation is observed as fixed only in December 2020, that is 3 months later.

**Figure 3:**
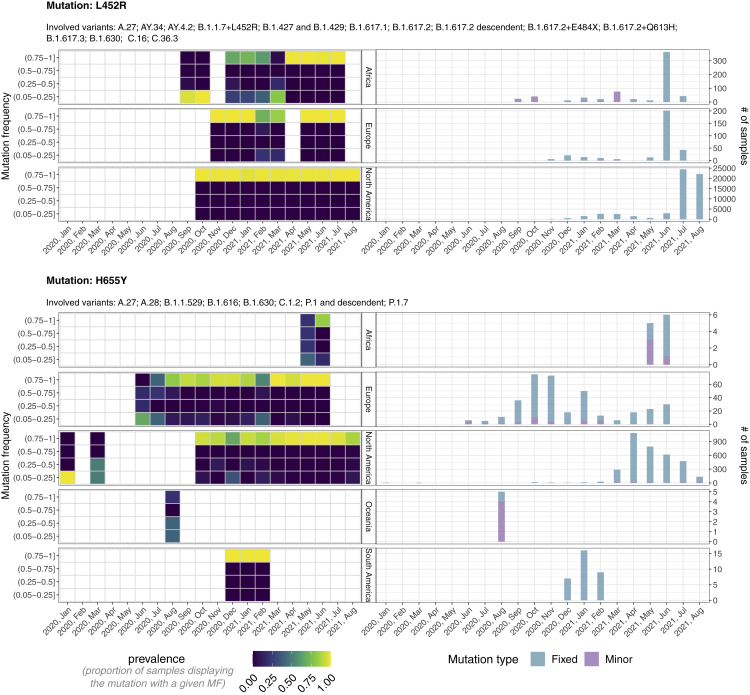
Mutant frequency and prevalence variation in time of SMoIs S:L452R and S:H655Y The leftmost panels return the distribution of the mutation frequency (MF) of all samples with SMoIs S:L452R (upper panels) and S:H655Y (lower), grouped by month and geographical region. Each cell shows the proportion of samples showing the mutation with that specific MF. The rightmost panels show the number of samples showing the mutations either as minor (MF ≥5% and <50%) or as fixed (MF ≥50%). The lineages associated to both variants are also displayed.

A similar trend is observed for mutation S:H655Y, which is firstly detected as minor in January 2020 in North America and as fixed in June 2020 in Europe (5 months in advance at the global scale) and in October 2020 in North America (9 months in advance at the local scale). Notice also that when mutation S:H655Y was first detected in Europe, Africa, and Oceania, the large majority of samples exhibited it as minor. These important results show that, once a variant is identified as VUM, VOI, or VOC, it is possible to detect the related mutations considerably before their fixation at both the global and the local scale, allowing for a timely implementation of containment strategies.

As an aside note, the distribution of the MF proves that most SMoIs are present either at a very low or at a very high frequency within hosts, suggesting the presence of strong purifying selection and of bottlenecks, as already observed in (Graudenzi et al., 2021) (see Figures S3–S36).

### Additional spike mutations

Even if a mutation is not associated to any of the known variants, it is possible to investigate its selection and fixation dynamics with remarkable anticipation by looking at its MF variation in time and its diffusion in the population.

To this end, we analyzed the list of (single-nucleotide) mutations meeting the following criteria: (1) falling on the spike gene, (2) not being associated to any of the variants of Table 1, (3) detected (with MF >5%) in at least 50 samples in the whole considered period, and (4) detected (with MF >5%) in at least 1% of the samples in the month in which detected with the highest prevalence. The final list includes 95 spike mutations (S:N30H was excluded from the analysis after manual curation).

**Figure 4:**
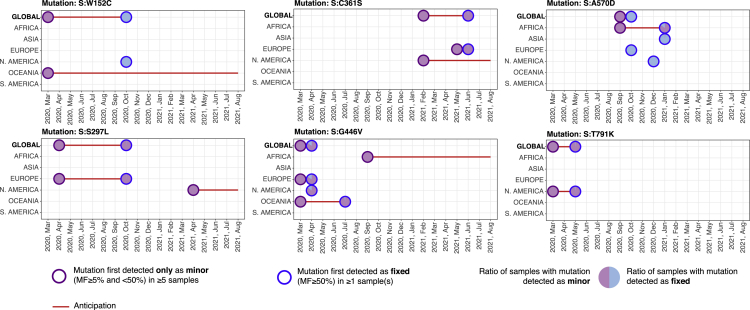
Early detection of 6 S mutations not associated to known variants Analysis of 6 S mutations originally detected as minor (in at least 5 samples) and only successively as fixed at the global scale, namely, S:W152C, S:S297L, S:C361S, S:G446V, S:A570D, and S:T791K. For further details, please refer to the caption of Figure 2. S mutations first detected as minor at the local scale are shown in Figure S1 in the supplementary information.

In brief, 6 (out of 95) mutations were initially found as minor at the global scale (S:W152C, S:S297L, S:C361S, S:G446V, S:A570D, S:T791K) (see Figure 4) and 11 additional mutations at the local scale (S:T95I, S:T167I, S:R682W, S:R685L, S:R685S, S:T716I, S:T791I, S:A892V, S:D1118H, S:G1124V, S:W1214G) (shown in Figure S1). Interestingly, mutation S:T95I is associated to Omicron variant, while not being listed as SMoI at the time of writing.

Also in this case, a significant anticipation is granted by the analysis of deep sequencing data, up to 7 months at the global and up to 17 months at the local scale, respectively.

Among the many mutations, S:V1264L was recently associated to Delta-1 variant and was hypothesized to underlie the outbreaks in Indonesia, Singapore, and Malaysia (see Yang. X.J. et al. EuropePMC preprint, 2021). Mutation S:W152C is associated to the lineage B.1.429 but not included in the list of SMoIs from any of the considered institutions. S:W1214G was identified as destabilizing in Jacob et al. (2021). These further results prove that the analysis of the minor mutation landscape might be effective to intercept hazardous S mutations prior to their fixation in the population, even when not associated to lists of known variants.

We finally note that 9 additional highly diffused S mutations were initially detected *both* as minor (in at least 5 samples) and fixed (in at least 1 sample) at the global scale (S:Q23H, S:A27S, S:S98F, S:F157L, S:L176F, S:K529M, S:T547I, S:G769V, S:V1264L), and other 6 S mutations in at least one geographical region (S:L5F, S:D80A, S:T95I, S:G446V, S:A892V, S:G1124V), demonstrating that relevant information might be missed by looking at consensus sequences only.

Mutation S:G446V shows particularly interesting dynamics, as it has been observed mostly (>90% samples) as minor mutation since March 2020, but has been showing an increase in MF since November 2020, and is now observed as a fixed variant approximately in 50% of the samples presenting the mutation in July 2021 and August 2021. Moreover, mutation S:G446V has been associated to attenuate monoclonal and serum antibody neutralization (Liu et al., 2021).

### Nucleocapsid mutations

We repeated the analysis by first focusing on the list of 13 NMoIs selected in Syed et al. (2021). Four of them, in particular, were associated to a significant increased mRNA delivery and expression from the analysis of SARS-CoV-2 virus-like particles (N:P199L, N:S202R, N:R203K, and N:R203M), and it was also hypothesized that one of such mutations may be responsible for the increased spread of variants including Delta (N:R203M). Furthermore, we selected an additional list of 83 highly diffused N mutations, with the criteria employed for the additional S mutations and described earlier (mutations N:D3E, N:D3H, N:D3V, and N:K256∗ were removed from the analysis after manual curation).

**Figure 5:**
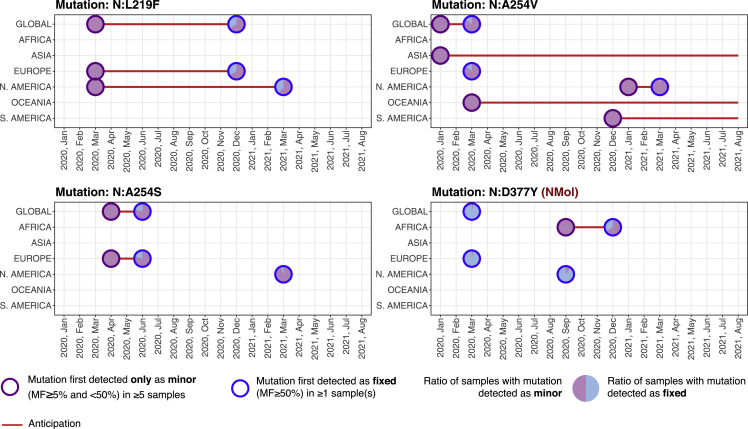
Early detection of N mutations Analysis of NMoI N:D377Y and of the three highly diffused *N* mutations originally detected as minor (in at least 5 samples) and only successively as fixed at the global scale, namely, N:L219F, N:A254S, and N:A254V. For further details please refer to the caption of Figure 2. N mutations first detected as minor at the local scale are shown in Figure S2 in the supplementary information.

As a result, 3 (out of 96) N mutations were initially found only as minor (in at least 5 samples) at the global scale (N:L219F, N:A254S, N:A254V), and 7 further mutations at the local scale (N:H145Y, N:S197L, N:G204A, N:L222M, N:Q244K, N:A305V, N:K374N), with distinct anticipation according to the cases (see Figure 5).

Of such mutations, N:D377Y was identified as NMoI in Syed et al. (2021) and in Africa was discovered as minor 3 months in advance. In Figure 5, one can find such mutation, in addition to three mutations first detected as minor at the global scale (see above), whereas the remaining mutations are shown in Figure S2. Although ad-hoc investigations on the possible functional effect of such mutations are clearly required, these findings demonstrate the effectiveness of deep sequencing data analyses to intercept possibly hazardous mutations.

In further support to this claim, let us also notice that 1 additional NMoI was initially detected *both* as minor (in at least 5 samples) and fixed (in at least 1 samples) (N:M234I) and 8 highly diffused N mutation (N:P13T, N:H145Y, N:R185C, N:P168S, N:G238C, N:E253D, N:S327L, N:D415G) at the global scale.

### Improved genomic surveillance of circulating variants

In addition to the early detection of mutations, deep sequencing data are important for the characterization of the intra-host diversity of SARS-CoV-2 samples that are already associated to circulating variants, overcoming the intrinsic limitations of studies on consensus sequences. This analysis has important repercussion in terms of genomic surveillance.

In fact, homoplastic minor mutations (i.e., retrieved in distant lineages, with sufficient sample size) are typically not related to transmission events but emerge independently in unrelated hosts, either due to a possible fitness advantage or due to mutational hotspots (Ramazzotti et al., 2021). Accordingly, they should be flagged and carefully considered, as they might possibly lead to the origination of new dangerous variants, if positively selected due to any underlying functional advantage. Thus, their characterization, in combination with analyses directed to the evaluation of positive selection processes (e.g., via molecular simulation—see the Limitations of the study Section for a dedicated discussion on the topic) might allow one to design opportune alert systems and timely intervention strategies.

**Figure 6:**
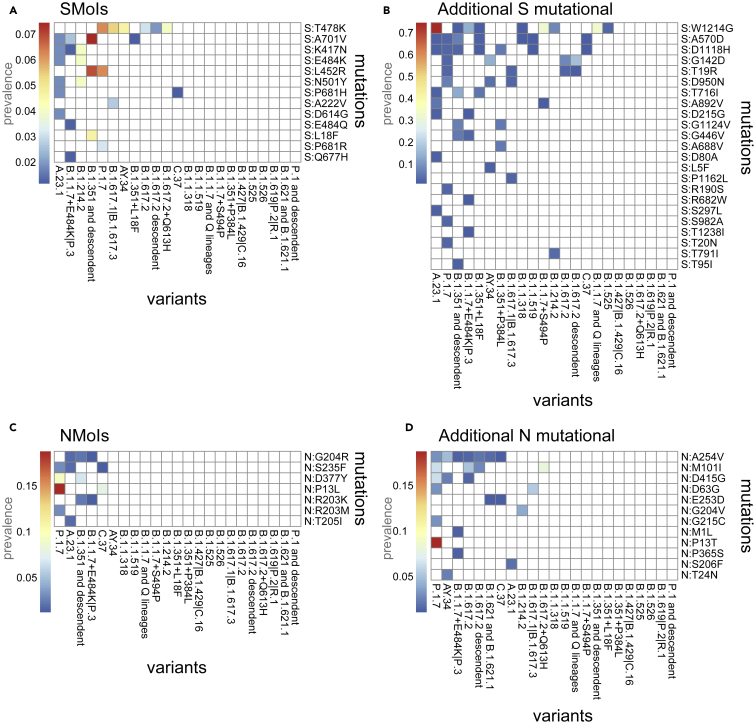
Analysis of homoplastic minor variants (A–D) The heatmaps show the prevalence (i.e., number of samples over the total) of the SMoIs (panel A), additional highly diffused S mutations (B), the NMoIs (C), and the additional highly diffused N mutations (D) retrieved as minor (MF >5% and ≤50%) in the samples associated to the variants of Table 1 via Pangolin (O’Toole et al., 2021a, 2021b). Only the mutations observed in at least 1% of the samples of any variant are shown.

We considered the list of mutations previously analyzed (35 SMoIs, 95 additional S mutations, 13 NMoIs and 83 additional N mutations) and assessed their presence in the minor state (MF ≥5% and <50%) in the samples associated via Pangolin (O’Toole et al., 2021a, 2021b) to the variants included in Table 1 (samples assigned to the “Other” category were excluded from the analysis, whereas, at the time of writing, Pangolin did not associate any sample to the Omicron variant). In Figure 6, one can find the prevalence of the selected minor mutations in the samples associated to the different variants. Only the mutations retrieved in at least 1% of the samples in at least one variant are considered.

In detail, 13 (out of 35) SMoIs are retrieved as minor and homoplastic (in at least 1% of the samples) and, in particular, 7 SMoIs are observed in more than one additional variant, namely S:T478K (in 6 variants), S:A701V (in 4), S:K417N (in 3), S:E484K (in 2), S:L452R (in 2), S:N501Y (in 2), and S:P681H (in 2). Twenty-three (out of 95) additional S mutations were found as homoplastic, with the most notable examples being mutations S:W1214G, S:A570D, S:D1118H, S:G142D, and S:T19R, which were found in at least 4 additional variants.

Similarly, 7 (out of 13) NMoIs were found as minor and homoplastic, with mutations N:G204R, N:S235F, N:D377Y, N:P13L, and N:R203K in at least 2 variants. Finally, 12 (out of 83) additional N mutations were detected in multiple variants, with mutations N:A254V, N:M101I, N:D415G, N:D63G, and N:E253D in at least 2 additional variants. This result points at a possible ongoing selection process, supporting the hypothesis of an important, yet underestimated, functional impact of mutations of this protein in SARS-CoV-2 evolution.

Overall, 19 (out of 44) variants listed in Table 1 (dark purple check marks) display homoplastic minor (S or N) mutations of interest. This result confirms the benefits of analyzing deep sequencing data to pinpoint the emergence of possibly hazardous mutations, also in samples harboring known variants, with an improved resolution with respect to standard analyses of consensus sequences.

## Discussion

Thanks to the largest up-to-date analysis of deep sequencing datasets of SARS-CoV-2 samples, we proved that standard studies based on consensus sequences might be scarcely effective for the early detection of mutations of interests and for the fine monitoring of homoplastic mutations that might lead to the origination of new variants. These aspects are even more relevant when considering the exceptional proportion of the COVID-19 pandemic and should be wisely considered in the ominous prospective of future epidemics.

Accordingly, a refined estimation of key epidemiological parameters (e.g., $R_{t}$) from deep sequencing data might lead to significant differences in the predictions delivered by the wide range of currently available epidemiological models (Davis et al., 2021; Kraemer et al., 2021), as well as in the accuracy and robustness of phylodynamics methods, which in most cases rely on consensus sequences (Duchene et al., 2020). This might affect, in turn, the geo-temporal narrative on variants origination, as well as that of infection chains and (multiple) introductions (Deng et al., 2020), possibly guiding improved testing and response strategies.

In this work, we started the analysis from the set of mutations related to known variants. Yet, a large set of additional S and N mutations were detected as minor with great advance with respect to standard analyses. As specified in the Limitations of the study section, it would be important to combine this data-driven result with automated approaches aimed at estimating the fitness advantage of mutations and variants.

Furthermore, the difference in the anticipation window related to the distinct geographical region suggests a straightforward way of improving current surveillance practices: once a variant start being monitored, even if already fixed in some areas, the analysis of the related minor mutations on different locations might allow one to intercept outbreak clusters with great advance, as well as to better estimate its overall prevalence.

It is also vital to point out that both the overall number of (minor) mutations detected in advance, and the magnitude of anticipation, would have dramatically benefited from the possibility of accessing a larger number of deep sequencing datasets, especially at the beginning of the epidemic. This issue mostly resulted from the absence of shared standards for testing and sequencing, which also contributed to the origination of relevant sampling biases and geographical inhomogeneities (Díaz-Pachón and Rao, 2021).

In this respect, given the strong evidences of our results, a methodological paradigm shift aimed at exploiting deep sequencing data seems to be opportune to improve genomic surveillance and might take advantage of the ever-increasing computational power and the pervasive data-sharing networks available to the scientific community. Accordingly, we advocate a collective effort for the definition of standardized best practices for deep sequencing data processing, analysis, and sharing (e.g., distribution of VCF files in FAIR-compliant repositories (Wilkinson et al., 2016)), to be implemented on top of existing databases and portals.

### Limitations of the study

Any analysis of intra-host viral diversity highly depends on the quality of upstream variant/haplotype calling, which is in turn closely related to the adopted technology and the testing criteria (Fuhrmann et al., 2021). When dealing with low-frequency mutations, one of the main criticisms lies in the difficulty of identifying true mutations from sequencing artifacts or phantom mutations, due, e.g., to mutational hotspots (Bandelt et al., 2002), as well as the possibility of dropouts, due, e.g., to uneven coverage (Posada-Cespedes et al., 2017). The analysis of distinct sets of minor variants might deceive statistical inference approaches, leading to partially incorrect results, and this might apply to the case of early detection of mutations as well. To this end, many variant callers currently exist to correct for data-specific errors (Knyazev et al., 2021) and might be tested to assess the robustness of the results discussed hereby.

It is also important to remark that the filtering criteria employed in the detection time analysis (Results Section) are—by construction—arbitrary, even if in this case they were designed to ensure a good trade-off between sensitivity of the analysis and overall statistical robustness. In fact, we decided to employ stringent criteria for minor mutations (e.g., we considered only the mutations observed as minor in at least 5 samples), so to reduce the impact of noisy observations and sequencing artifacts, and much softer criteria for fixed mutations (e.g., we considered such mutations when found in at least 1 sample), so to demonstrate the general validity of our approach. Even if the exploration of the countless filtering combinations is unpractical, we expect that different and less strict criteria might lead to even greater magnitudes of anticipation in the detection of mutations, further confirming our results.

Another general limitation of this study is related to the practical unfeasibility of providing up-to-date results with respect to the current (and ever-changing) knowledge regarding the evolution of the SARS-CoV-2 virus and its variants, which however does not affect the general message of the work. For updates on SARS-CoV-2 phylogenomic model and variants, we refer the reader to the websites of public bodies such as World Health Organization, 2021; Centers for Disease Control and Prevention, 2021; and European Centre for Disease Prevention and Control, 2021 or to portals such as Shu and McCauley, 2017; Hadfield et al., 2018; O’Toole et al., 2021a, 2021b; National Center for Biotechnology Information, 2021; and EMBL-EBI Covid-19 Data Portal, 2021 (Hodcroft, 2021).

As mentioned in the previous section, several advanced methods for the investigation of the selection processes involving genomic mutations are currently available, e.g., via molecular simulations of the functional effects of genomic changes, and represent a fundamental complementary aspect of genomic surveillance. For instance, in Starr et al. (2020), the authors evaluate the fitness of SARS-CoV-2 mutations by estimating their effect on: (1) the human ACE2 receptor binding affinity and (2) the virus receptor binding domain (RBD) folding stability, thus providing a further instrument for vaccine design and genomic surveillance. In Pond (2021), state-of-the-art statistical methods are employed to identify which positions in the SARS-CoV-2 genome may be subject to positive or negative selection, also allowing one to pinpoint the most interesting sites according to different criteria, via an interactive tool. Further studies aimed at predicting the fitness advantage of mutations are presented, e.g., in Shang et al., 2020; Yan et al., 2020; Wu et al., 2021; Zhou et al., 2021; and Greaney et al., 2021. It would be surely worthwhile to integrate similar approaches with methods for the early detection of mutations from deep sequencing data, as proposed here, so to deliver comprehensive genomic surveillance systems covering the many facets of viral evolution.

We finally note that the limitations related to partial and inhomogeneous testing/sampling of the population are discussed in the Discussion section.

## STAR★Methods

### Key resource table

**Table:** 

REAGENT or RESOURCE	SOURCE	IDENTIFIER
Software and algorithms		

BWA-MEM 0.7.17-r1188	Li, Heng, and Richard Durbin. “Fast and accurate short read alignment with Burrows–Wheeler transform.” Bioinformatics 25.14 (2009): 1754–1760.	http://bio-bwa.sourceforge.net/bwa.shtml
Samtools 1.10	Li, Heng. “Improving SNP discovery by base alignment quality.” Bioinformatics 27.8 (2011): 1157–1158.	http://samtools.sourceforge.net/
iVar 1.3.1	Grubaugh, Nathan D. et al. “An amplicon-based sequencing framework for accurately measuring intra-host virus diversity using PrimalSeq and iVar.” Genome biology 20.1 (2019): 1–19.	https://andersen-lab.github.io/ivar/html/manualpage.html
The R Project for Statistical Computing	Team, R. Core. “R: A language and environment for statistical computing.” (2013): 201.	https://www.r-project.org
Custom code to replicate the analyses presented in the text.	This paper.	https://github.com/BIMIB-DISCo/SARS-CoV-2-early-detection

### Resource availability

#### Lead contact

Further information and requests for resources and reagents should be directed to and will be fulfilled by the Lead Contact: Alex Graudenzi, Dept. of Informatics, Systems and Communication, University of Milan-Bicocca, Viale Sarca 336, 20126, Milan, Italy. alex.graudenzi@unimib.it

#### Materials availability

This study did not generate new unique reagents.

### Methods details

#### Datasets

We analyzed a total 391,173 samples from distinct individuals obtained from 137 public NCBI BioProjects (see Table S1 for the list of BioProjects and samples metadata). All selected FASTQ samples were paired-end amplicon sequencing data prepared following the COVID-19 ARTIC v3 Illumina library construction and sequencing protocol.

#### Mutation calling

Mutation calling was performed by employing the iVar (version 1.3.1) (Grubaugh et al., 2019) recommended pipeline to analyze SARS-CoV-2 ARTIC v3 amplicon sequencing data, with quality check with default parameters (minimum quality score threshold to count base =20). In particular, we performed the following steps: 1) FASTQ files were mapped to the reference genome SARS-CoV-2-ANC (Ramazzotti et al., 2021; Graudenzi et al., 2021) with *bwa mem* (version 0.7.17-r1188) (Li and Durbin, 2010). 2) Sorted BAM files were generated from *bwa mem* results using SAMtools (version 1.10) (Li et al., 2009). 3) ARTIC v3 primer sequences were trimmed using *ivar trim* command. 4) Trimmed sorted BAM files were built and indexed with SAMtools. 5) Mutation calling was performed from trimmed sorted BAM files using *ivar variants*, after applying SAMtools *mpileup*. 6) Finally, *samtools depth* was used to extract coverage information from trimmed sorted BAM files.

#### Quality control

Quality control was performed on the mutations obtained with *iVar variants*. First, we selected the samples with a coverage of at least 100 reads over at least 90% of the viral genome. Then, we performed further filtering by selecting only mutations with variant frequency of at least 5%, coverage of at least 100 and p-value resulting from *ivar variants* less than 0.01. Finally, samples with more than 100 mutations (after filtering) were removed, to obtain a final dataset comprising a total 220,788 samples and 7,855,379 (88,889 unique) mutations (see Table S1).

### Quantification and statistical analysis

#### Amino acid sequence annotation

We considered all nonsynonymous substitutions (i.e., we kept only single base mutations) and annotated them to the related amino acid sequence. To avoid ambiguities, we removed mutations spanning mismatching positions between SARS-CoV-2-ANC (Ramazzotti et al., 2021; Graudenzi et al., 2021) and other proposed SARS-CoV-2 reference genomes (Andersen et al., 2020; Bastola et al., 2020), namely positions 8782, 28144 and 29095 of SARS-CoV-2-ANC were removed. This led us to a total of 4,962,209 and 46,903 unique amino acid changes (see Table S2).

#### Pangolin analysis

We created consensus sequences as input to Pangolin (O’Toole et al., 2021a, 2021b) from the mutations obtained from deep sequencing data as explained in *Mutation calling*. We considered mutations with MF ≥0.50, i.e., the standard consensus sequences as uploaded, e.g., on GISAID (Shu and McCauley, 2017). We created consensus sequences for each sample by adding to the reference genome SARS-CoV-2-ANC (Ramazzotti et al., 2021; Graudenzi et al., 2021) sequence, the substitutions, insertions, and deletions observed in the sample for each position and by choosing the one at higher MF if multiple mutations were detected in the same position. On such inputs, Pangolin was executed with default parameters and version v1.2.81.

## Data Availability

• SARS-CoV-2 paired-end Illumina Amplicon data are publicly available from NCBI National Center for Biotechnology Information (2021). The full list of analyzed samples is provided as Table S1. • The variant calling pipeline to analyze SARS-CoV-2 paired-end Illumina Amplicon sequencing data and the R script to perform amino acid annotation for nonsynonymous mutations are available on GitHub at: https://github.com/BIMIB-DISCo/SARS-CoV-2-early-detection. The code was also deposited at Zenodo and is publicly available as of the date of publication with https://doi.org/10.5281/zenodo.6566256. • Any additional information required to reanalyze the data reported in this paper is available from the lead contact upon request.

## Supplemental information

Document S1. Figure S1–S36

Table S1. The file contains the list of BioProjects and samples metadata of the datasets employed in the study, related to STAR Methods

Table S2. The file contains the annotated list of unique amino acid changes identified in the samples analyzed in this work, related to STAR Methods
