# Supplementary material for: Early detection and improved genomic surveillance of SARS-CoV-2 variants from deep sequencing data
Source: iScience. 2022 May 30;25(6):104487. doi: 10.1016/j.isci.2022.104487 (PMC9162787; doi:10.1016/j.isci.2022.104487)
Supplement: Document S1. Figure S1–S36 [file mmc1.pdf]

iScience, Volume *25*

Supplemental information

Early detection and improved genomic surveillance of SARS-CoV-2 variants from deep sequencing data

Daniele Ramazzotti, Davide Maspero, Fabrizio Angaroni, Silvia Spinelli, Marco Antoniotti, Rocco Piazza, and Alex Graudenzi

Supplementary Information: Early detection and improved genomic surveillance of SARS-CoV-2 variants from deep sequencing data

Daniele Ramazzotti[1,*], Davide Maspero[2,3], Fabrizio Angaroni[2], Silvia Spinelli[1]
Marco Antoniotti[2,4], Rocco Piazza[1,4], Alex Graudenzi[2,3,4,*]

[1] Dept. of Medicine and Surgery, Univ. of Milan-Bicocca, Milan

[2] Dept. of Informatics, Systems and Communication, Univ. of Milan-Bicocca, Milan

[3] Inst. of Molecular Bioimaging and Physiology,
Consiglio Nazionale delle Ricerche (IBFM-CNR), Segrate, Milan

[4] Bicocca Bioinformatics Biostatistics and Bioimaging Centre – B4, Milan

*To whom correspondence should be addressed;
e-mails: alex.graudenzi@unimib.it, daniele.ramazzotti@unimib.it

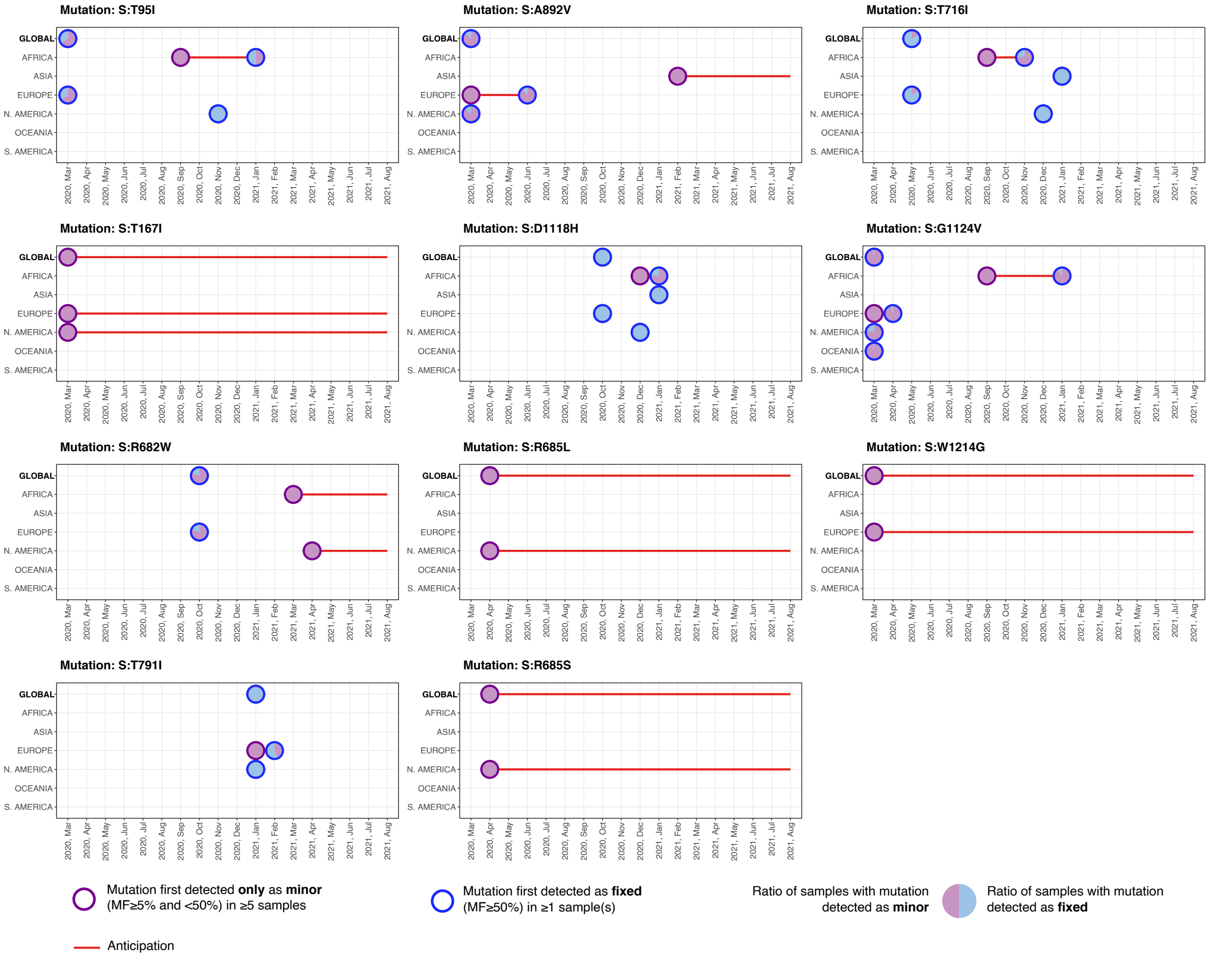

Figure S 1: **Early detection of 11 highly diffused S mutations from deep sequencing data. Related to Figures 2, 4, 5.** Analysis of S mutations: S:T95I, S:T167I, S:R682W, S:R685L, S:R685S, S:T716I, S:T791I, S:A892V, S:D1118H, S:G1124V, S:W1214G. Circles with *purple* borders mark the first month in which the mutation was detected as minor (MF $\geq$ 5% and $<$ 50%) in at least 5 samples, while been still undetected as fixed (MF $\geq$ 50%); circles with *blue* borders mark the month in which the mutation was first detected as fixed in at least 1 sample; red lines highlight the anticipation (when $>$ 1 months). The analysis is performed by splitting the samples in the 6 distinct geographical regions and by reporting the corresponding results at the global scale. All circles contain a pie-chart that displays the ratio of samples showing that mutation either as minor or fixed in that month (further details are provided in the main text). For each SMoI the related variants are also reported.

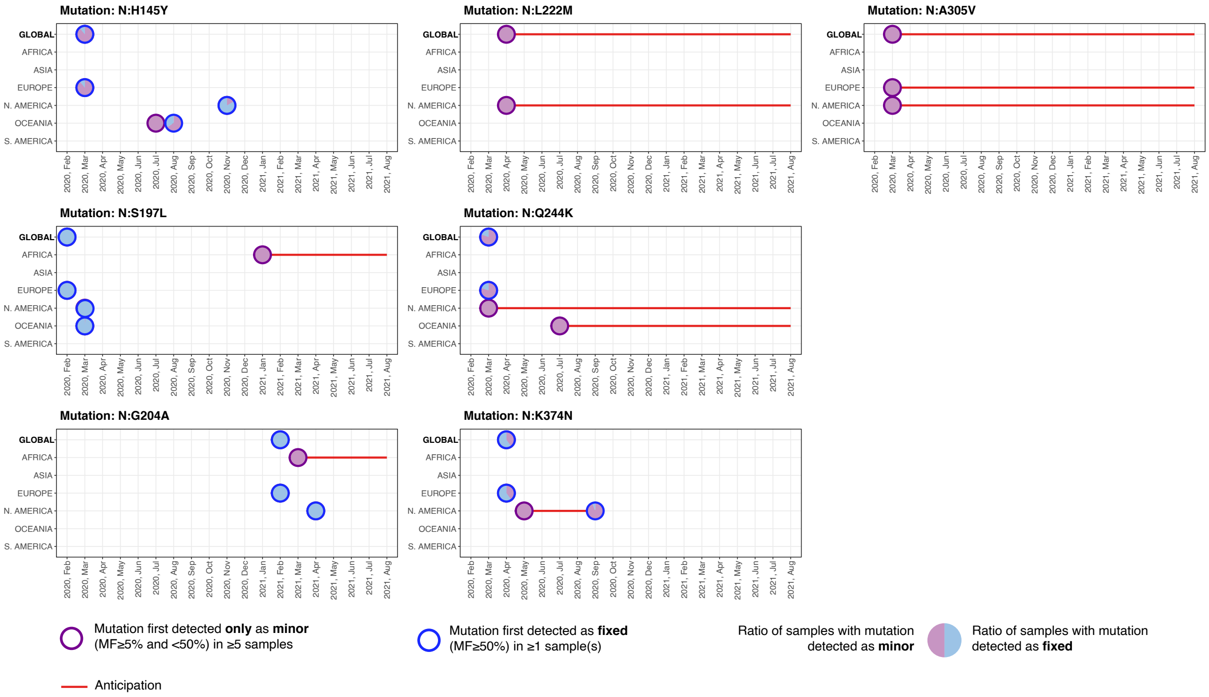

Figure S 2: **Early detection of 7 highly diffused N mutations from deep sequencing data. Related to Figures 2, 4, 5.** Analysis of N mutations: N:H145Y, N:S197L, N:G204A, N:L222M, N:Q244K, N:A305V, N:K374N. Circles with *purple* borders mark the first month in which the mutation was detected as minor (MF $\geq 5\%$ and $< 50\%$) in at least 5 samples, while been still undetected as fixed (MF $\geq 50\%$); circles with *blue* borders mark the month in which the mutation was first detected as fixed in at least 1 sample; red lines highlight the anticipation (when > 1 months). The analysis is performed by splitting the samples in the 6 distinct geographical regions and by reporting the corresponding results at the global scale. All circles contain a pie-chart that displays the ratio of samples showing that mutation either as minor or fixed in that month (further details are provided in the main text). For each SMoI the related variants are also reported.

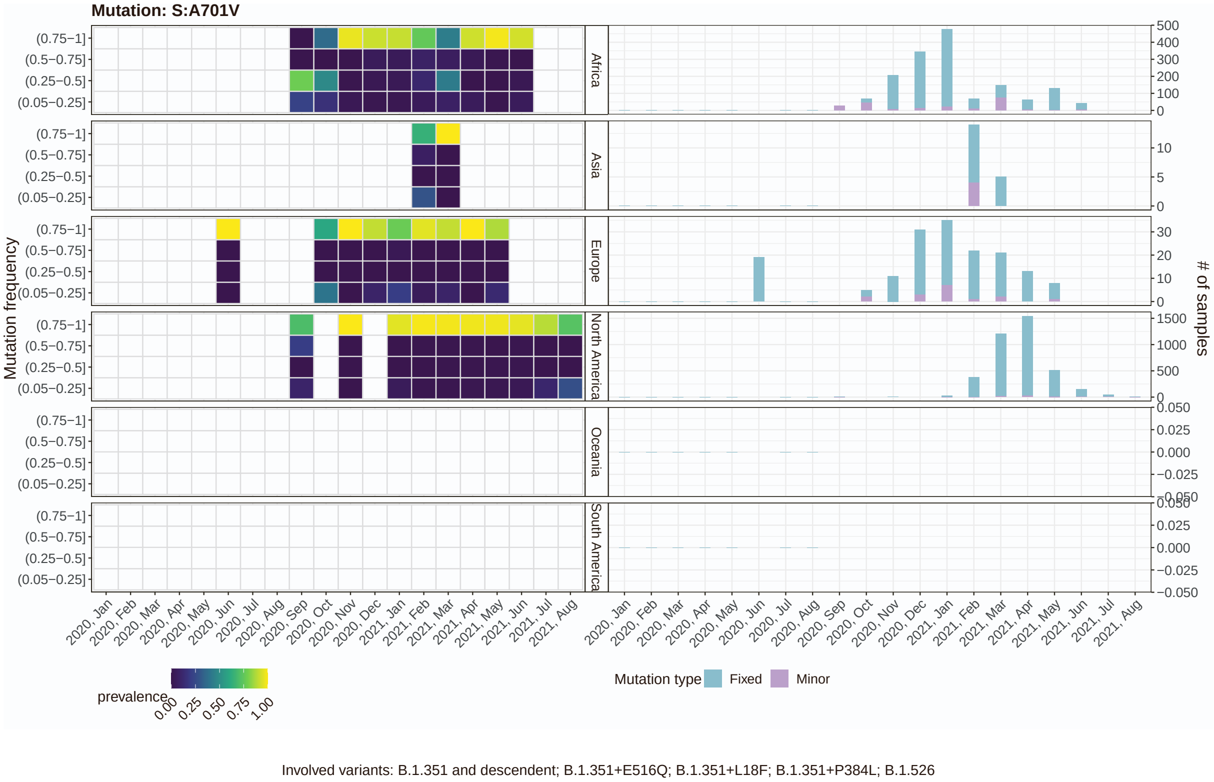

Figure S 3: **Mutant frequency and prevalence variation in time of mutation: S:A701V. Related to Figure 3.** The leftmost panels return the distribution of the mutation frequency (MF) of all samples with the mutation, grouped by month and geographical region. Each cell shows the proportion of samples showing the mutation with that specific MF. The rightmost panels show the number of samples showing the mutations either as minor (MF $\geq$ 5% and $<$ 50%) or fixed (MF $\geq$ 50%).

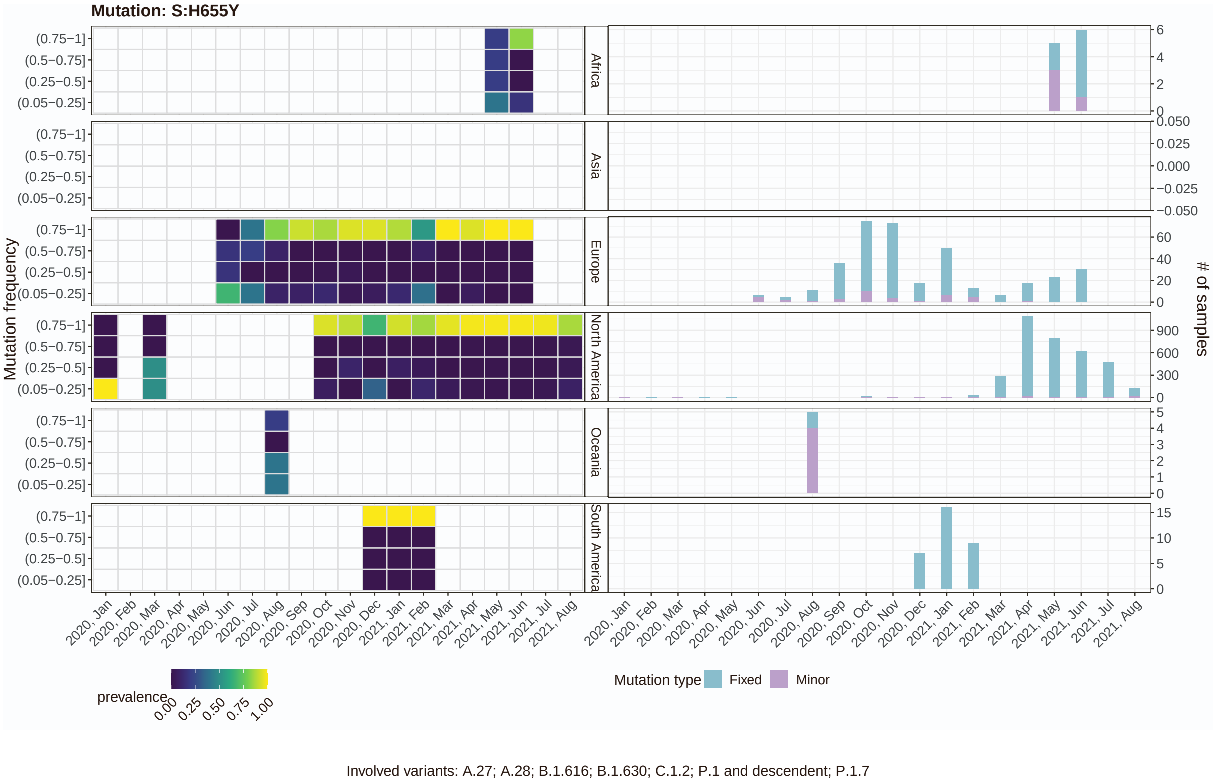

Figure S 4: **Mutant frequency and prevalence variation in time of mutation: S:H655Y. Related to Figure 3.** Please refer to the caption of Figure S 3

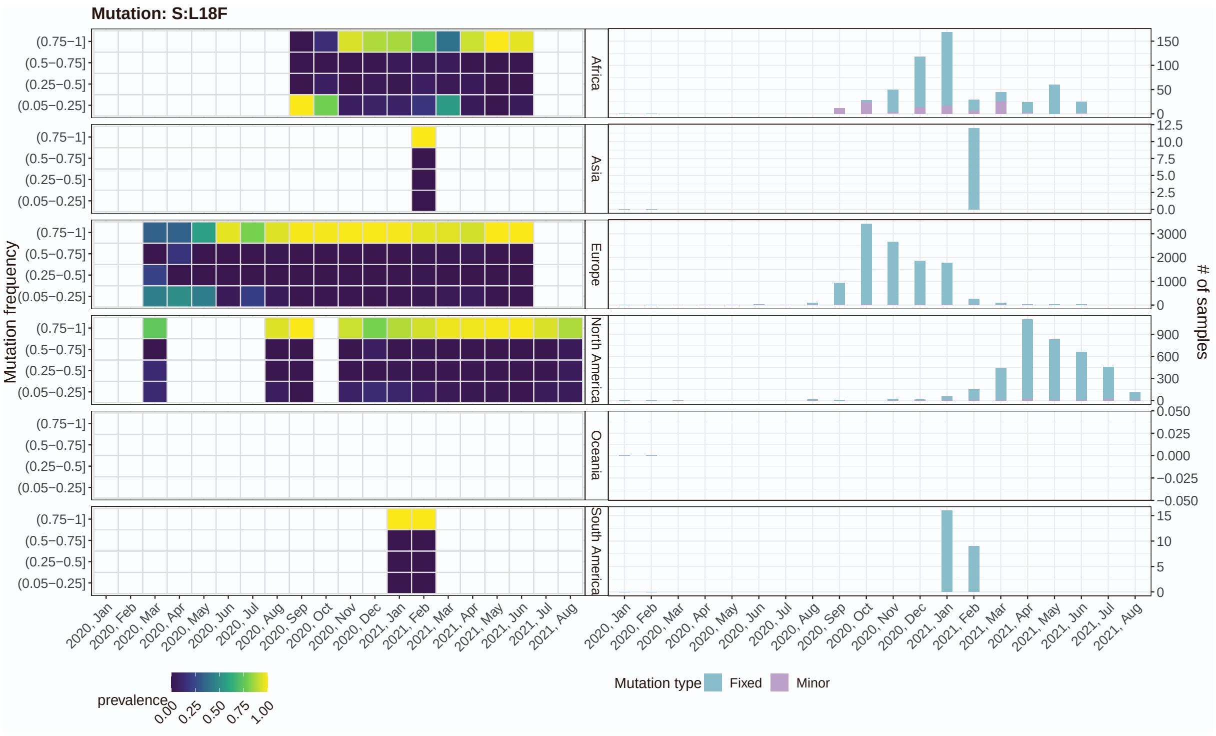

Figure S 5: **Mutant frequency and prevalence variation in time of mutation: S:L18F. Related to Figure 3.** Please refer to the caption of Figure S 3

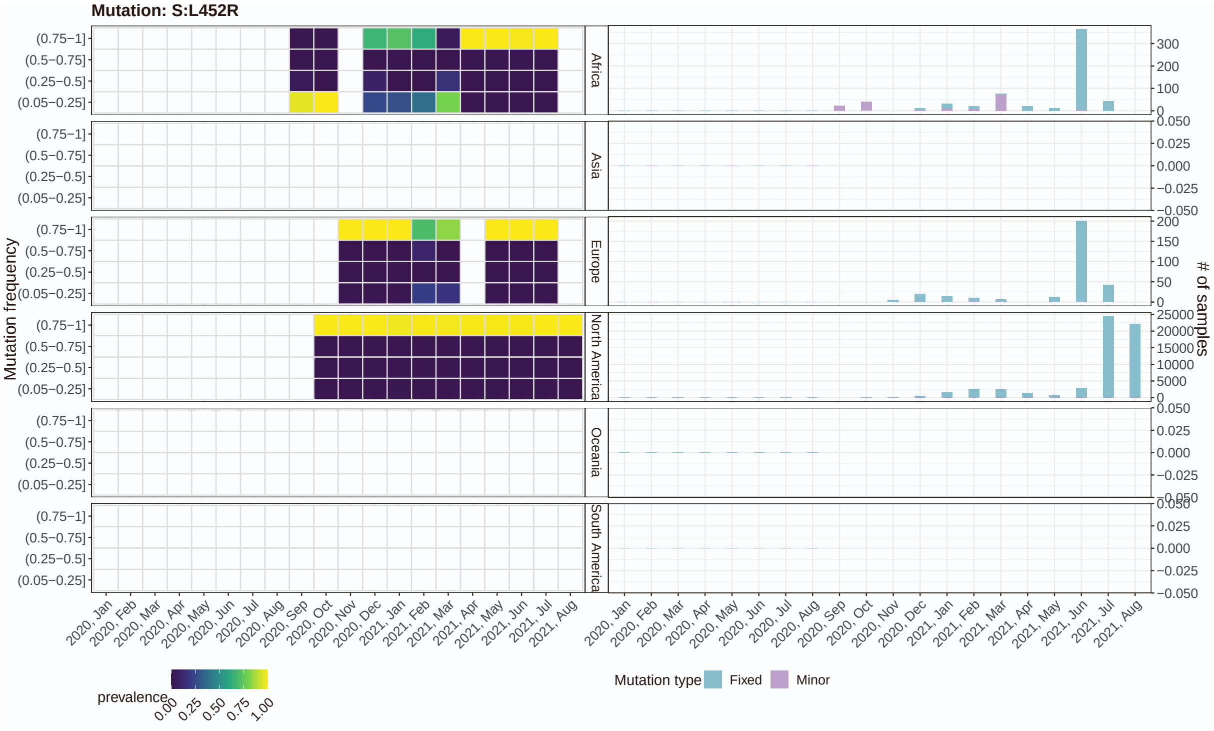

Involved variants: A.27; AY.34; AY.4.2; B.1.1.7+L452R; B.1.427|B.1.429|C.16; B.1.617.1|B.1.617.3; B.1.617.2; B.1.617.2 descendent; B.1.617.2+E484X; B.1.617.2+Q613H; B.1.630 C.36.3

Figure S 6: **Mutant frequency and prevalence variation in time of mutation: S:L452R. Related to Figure 3.** Please refer to the caption of Figure S 3

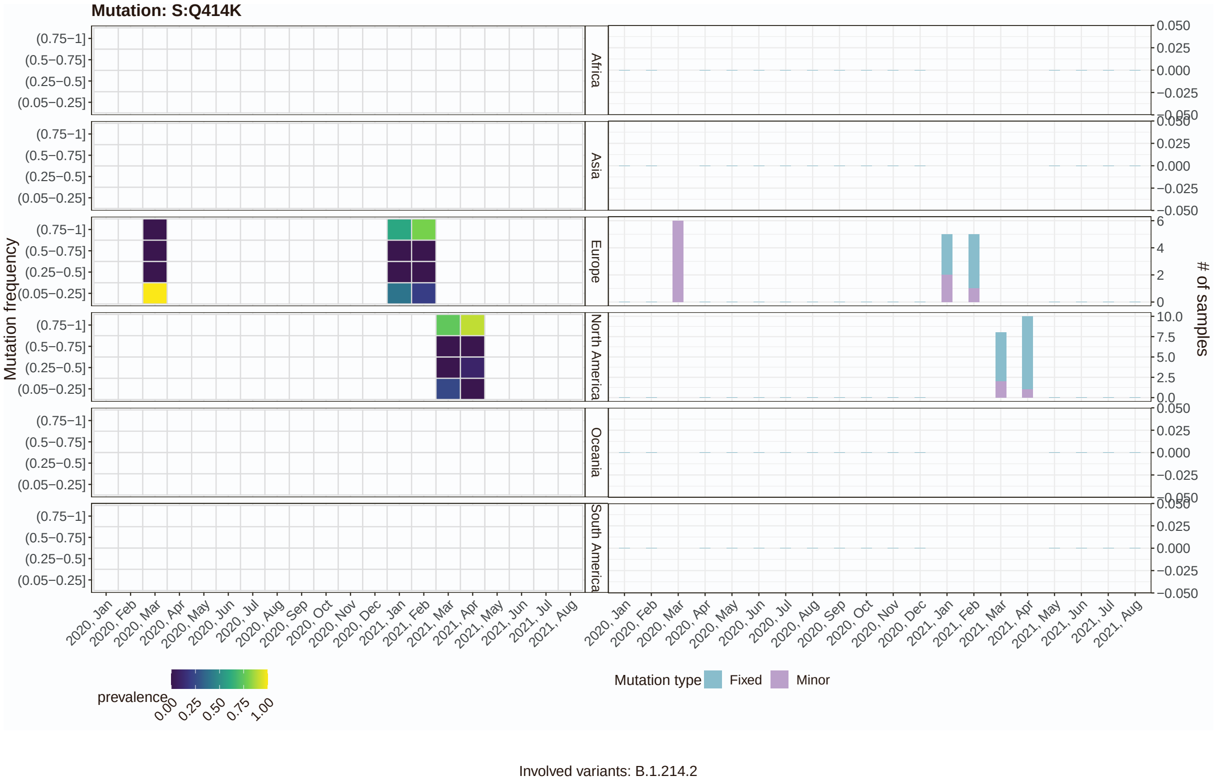

Figure S 7: **Mutant frequency and prevalence variation in time of mutation: S:Q414K. Related to Figure 3.** Please refer to the caption of Figure S 3

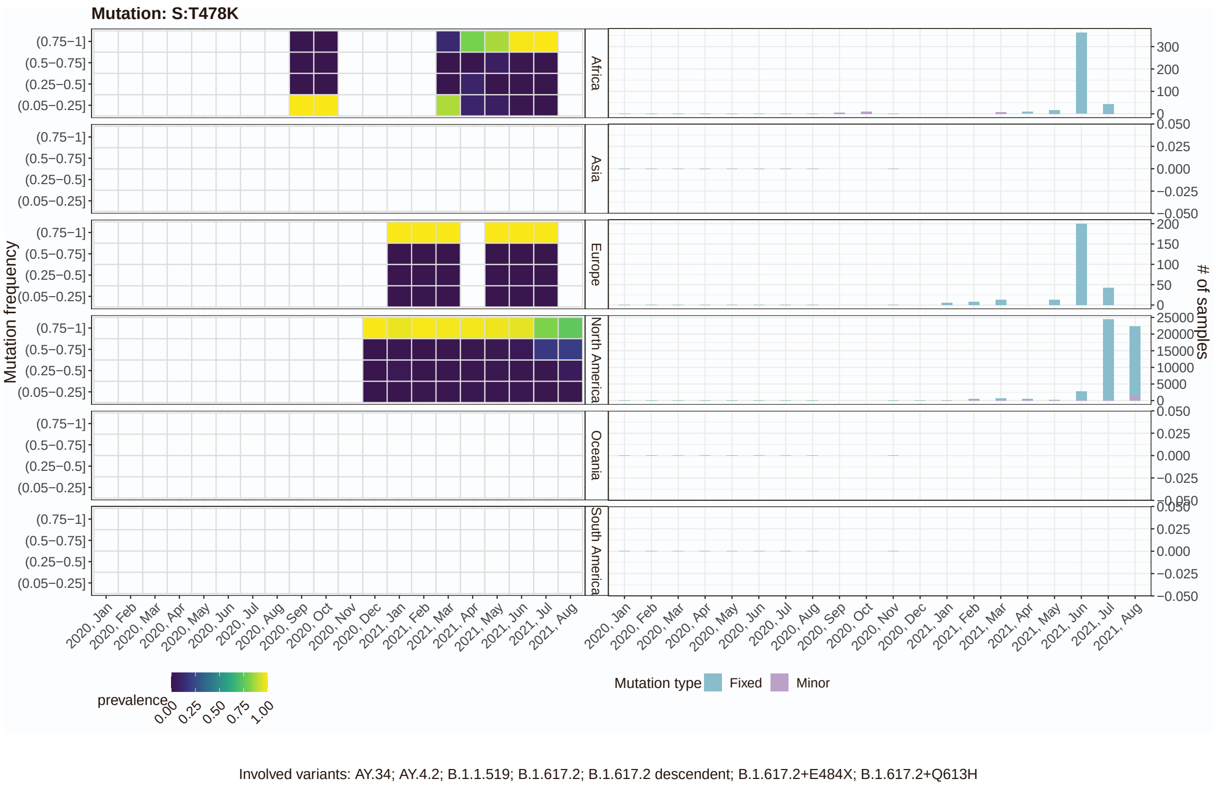

Figure S 8: **Mutant frequency and prevalence variation in time of mutation: S:T478K. Related to Figure 3.** Please refer to the caption of Figure S 3

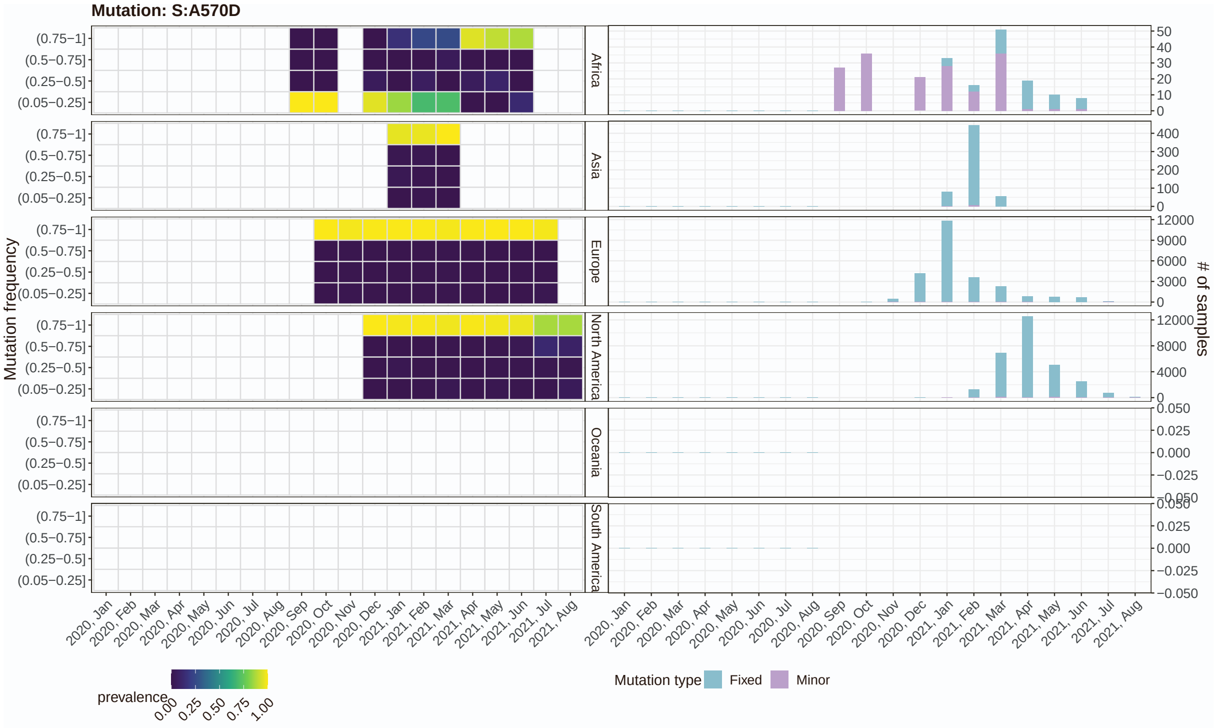

Figure S 9: **Mutant frequency and prevalence variation in time of mutation: S:A570D. Related to Figure 3.** Please refer to the caption of Figure S 3

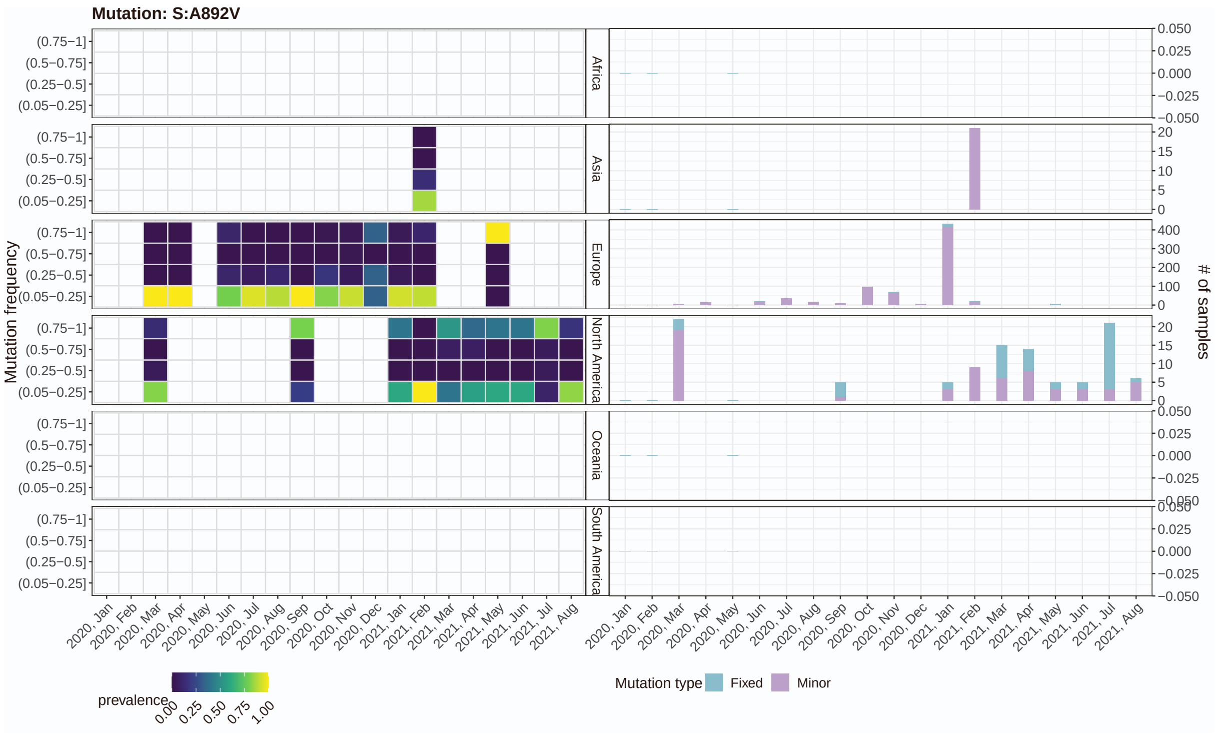

Figure S 10: **Mutant frequency and prevalence variation in time of mutation: S:A892V. Related to Figure 3.** Please refer to the caption of Figure S 3

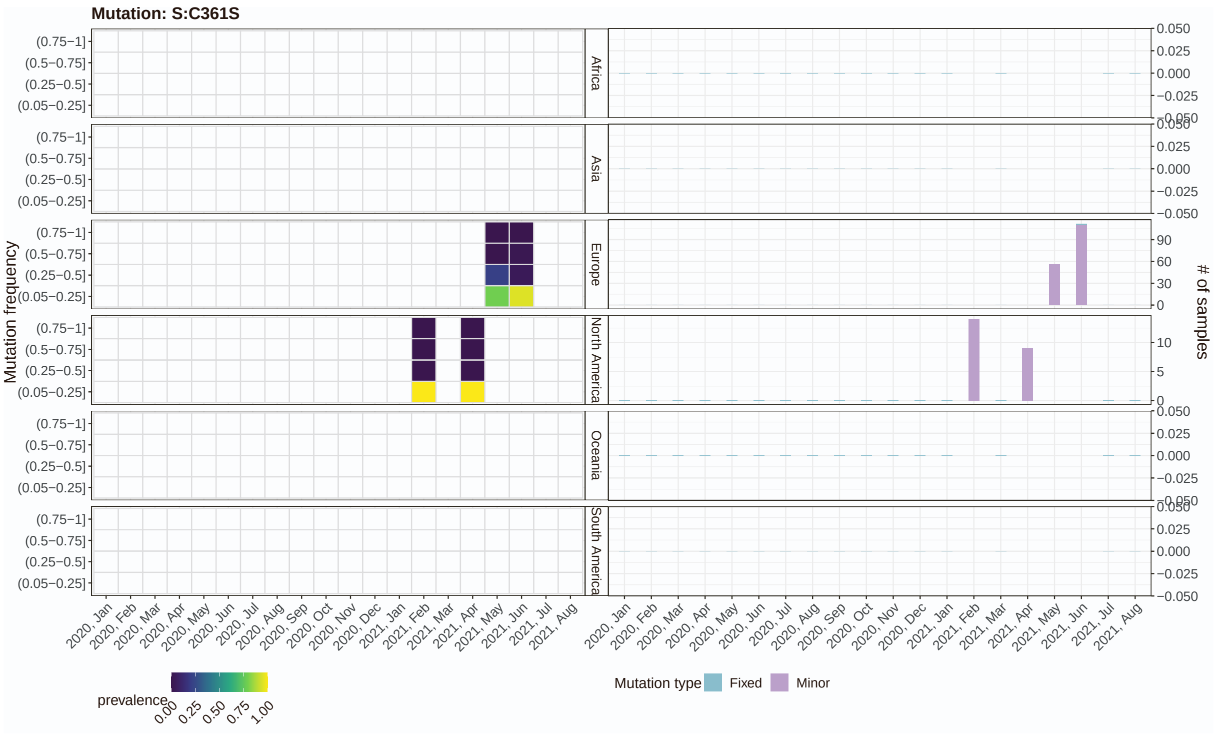

Figure S 11: **Mutant frequency and prevalence variation in time of mutation: S:C361S. Related to Figure 3.** Please refer to the caption of Figure S 3

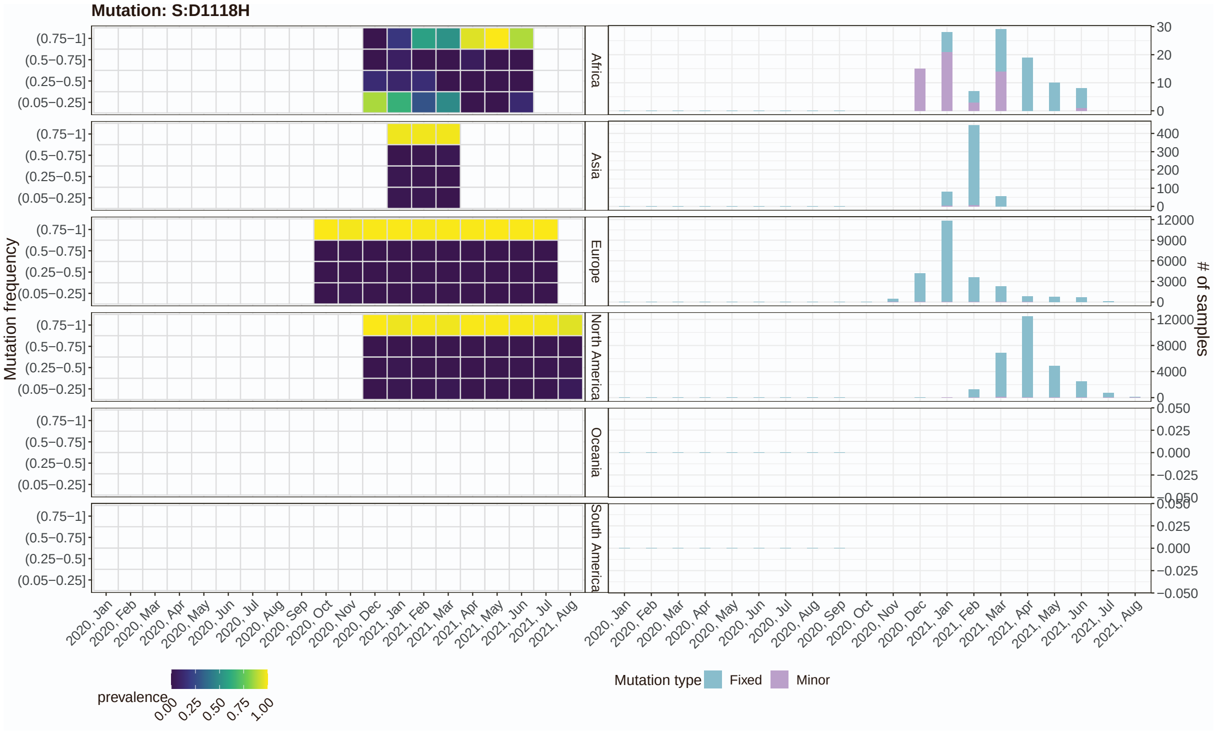

Figure S 12: **Mutant frequency and prevalence variation in time of mutation: S:D1118H. Related to Figure 3.** Please refer to the caption of Figure S 3

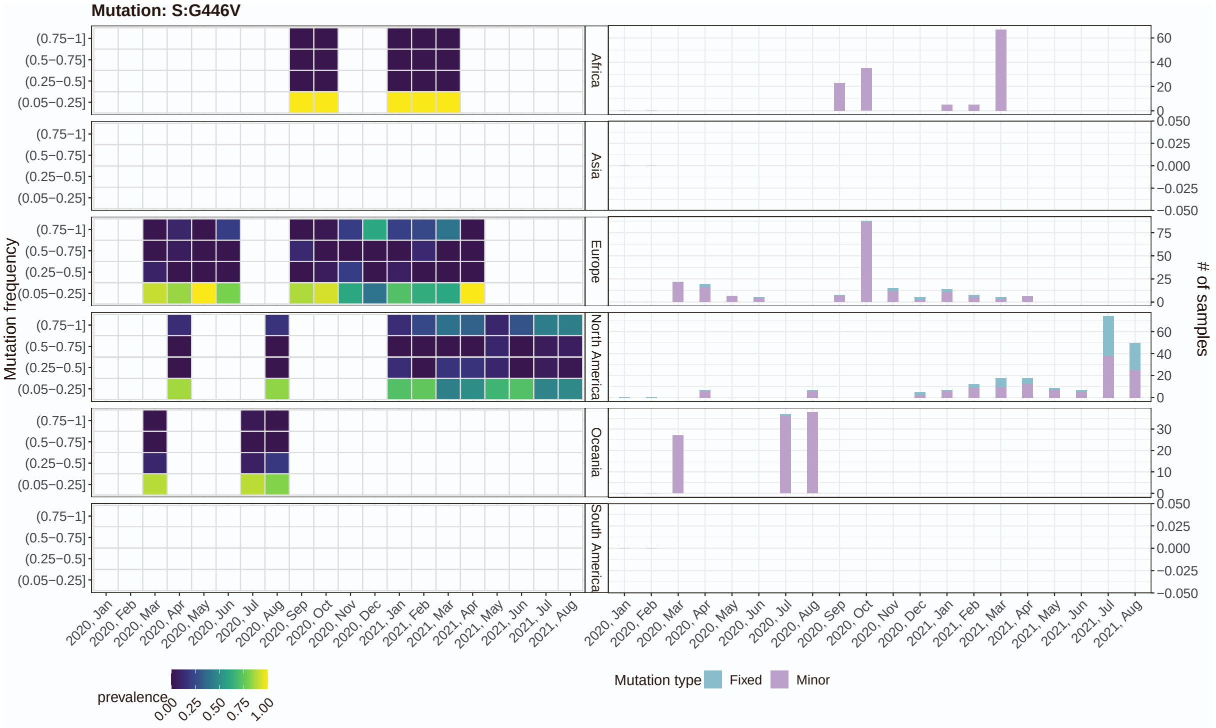

Figure S 13: **Mutant frequency and prevalence variation in time of mutation: S:G446V. Related to Figure 3.** Please refer to the caption of Figure S 3

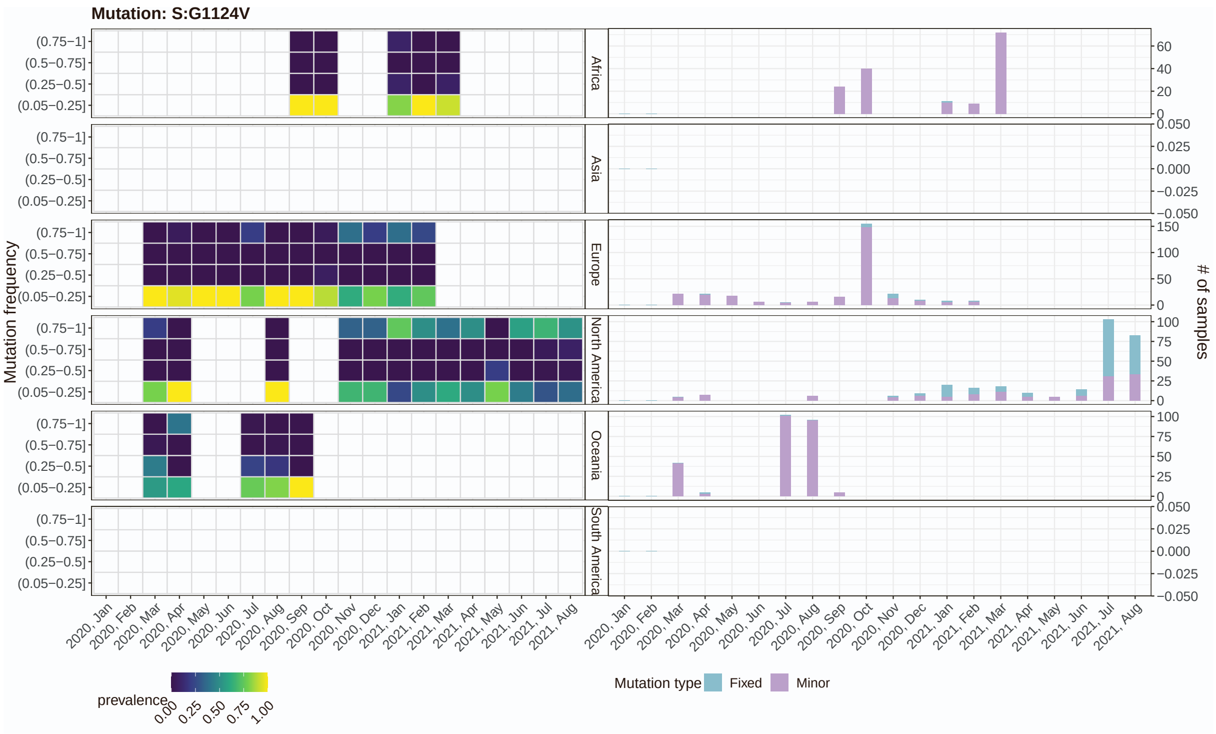

Figure S 14: **Mutant frequency and prevalence variation in time of mutation: S:G1124V. Related to Figure 3.** Please refer to the caption of Figure S 3

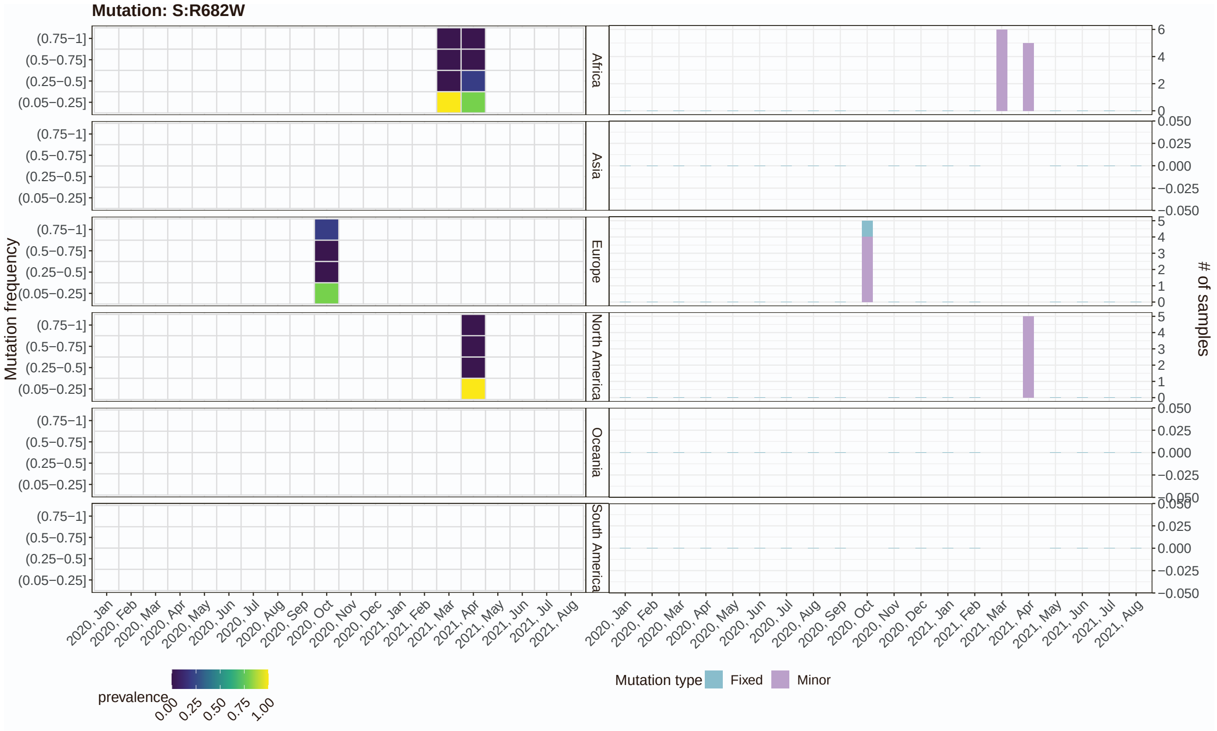

Figure S 15: **Mutant frequency and prevalence variation in time of mutation: S:R682W. Related to Figure 3.** Please refer to the caption of Figure S 3

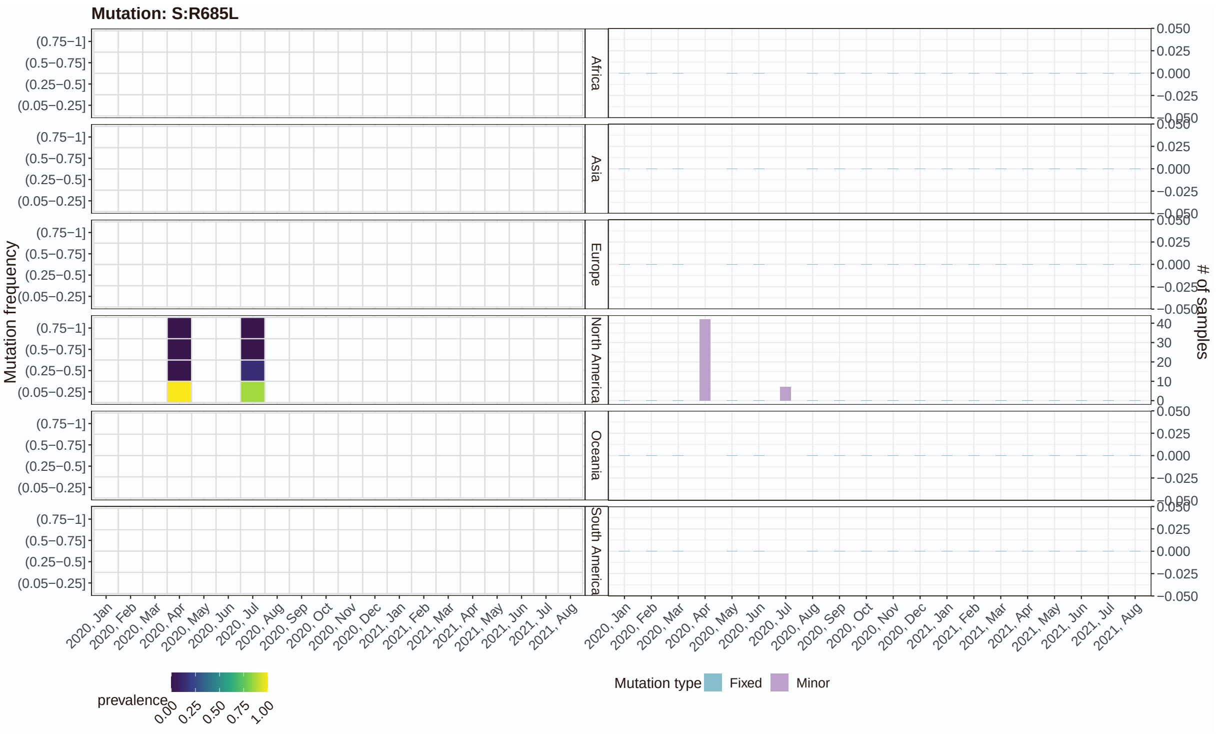

Figure S 16: **Mutant frequency and prevalence variation in time of mutation: S:R685L. Related to Figure 3.** Please refer to the caption of Figure S 3

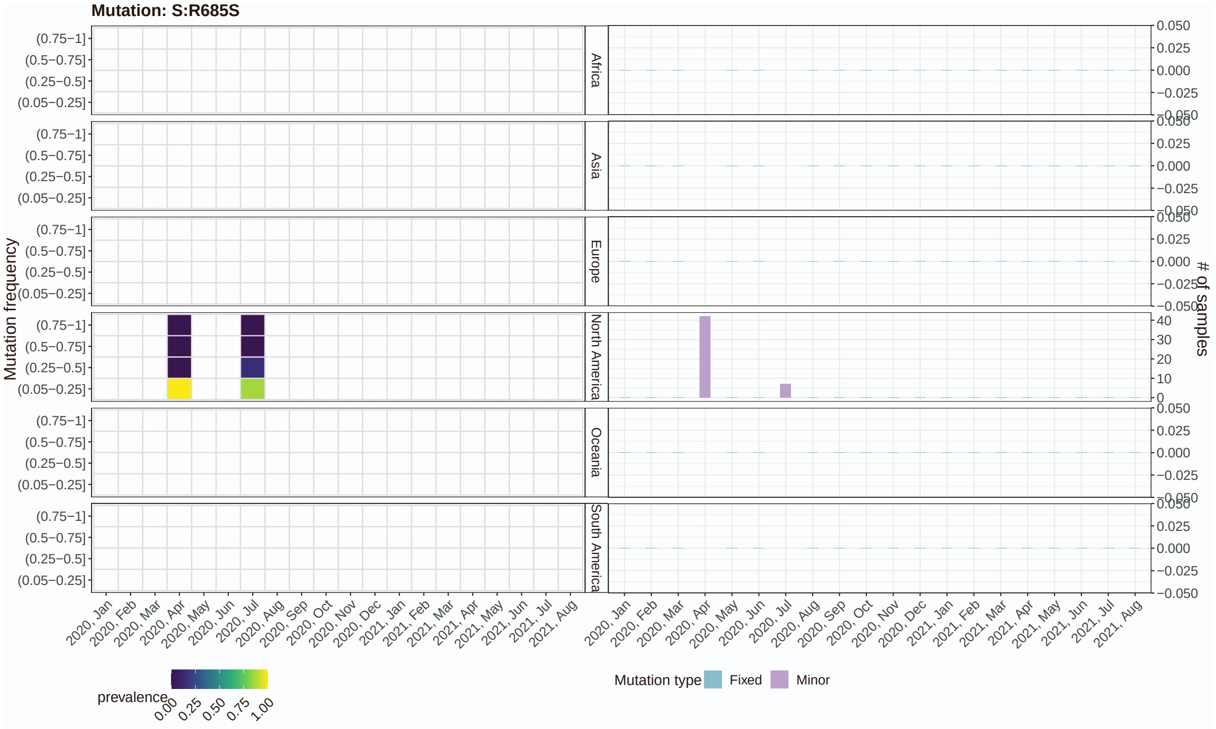

Figure S 17: **Mutant frequency and prevalence variation in time of mutation: S:R685S. Related to Figure 3.** Please refer to the caption of Figure S 3

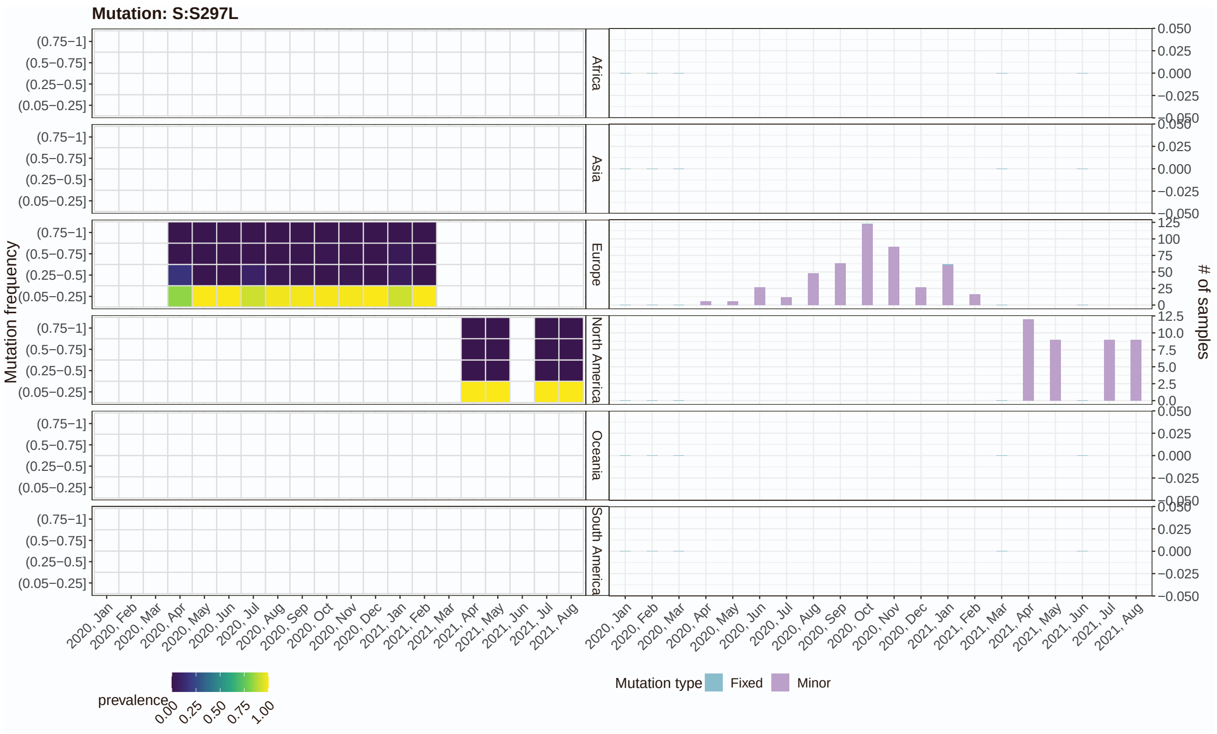

Figure S 18: **Mutant frequency and prevalence variation in time of mutation: S:S297L. Related to Figure 3.** Please refer to the caption of Figure S 3

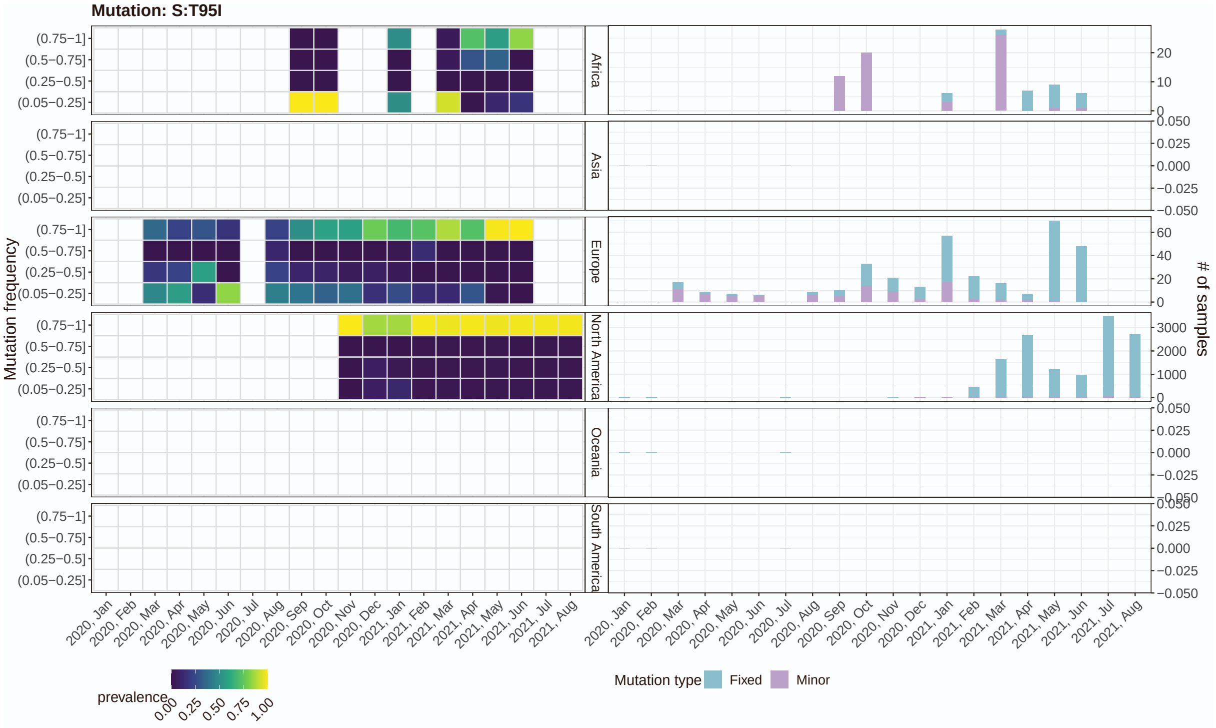

Figure S 19: **Mutant frequency and prevalence variation in time of mutation: S:T95I. Related to Figure 3.** Please refer to the caption of Figure S 3

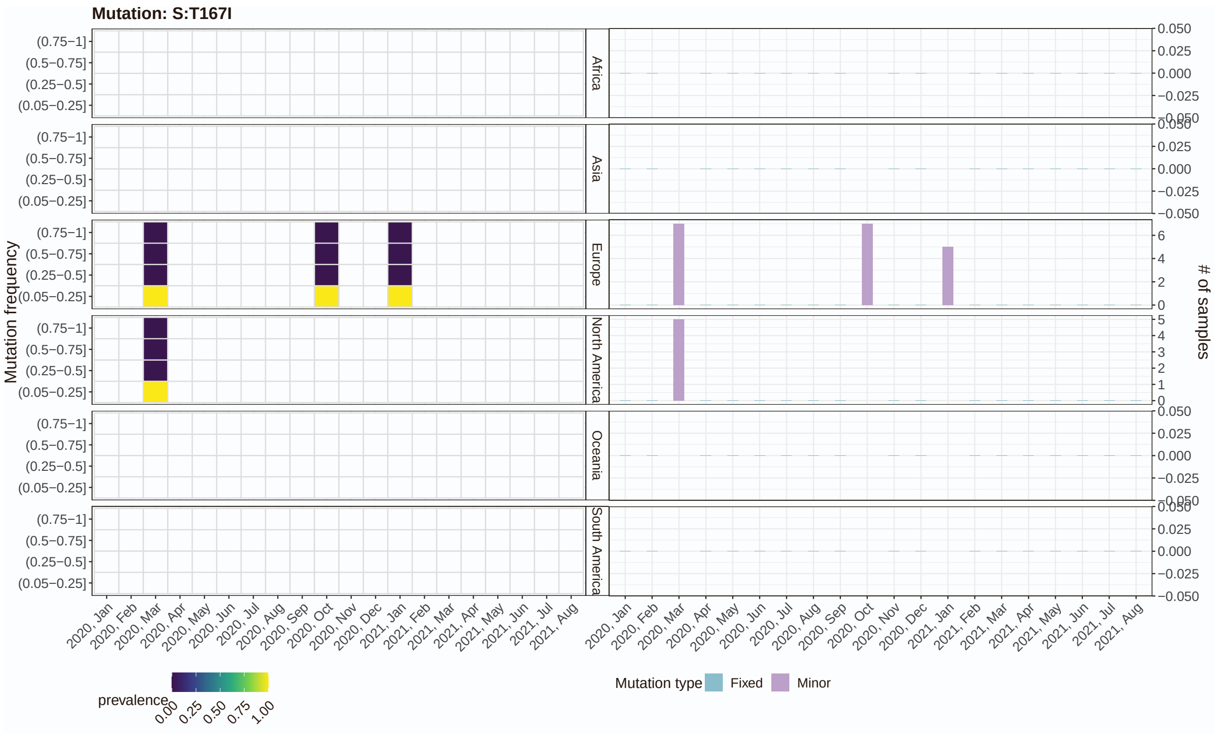

Figure S 20: **Mutant frequency and prevalence variation in time of mutation: S:T167I. Related to Figure 3.** Please refer to the caption of Figure S 3

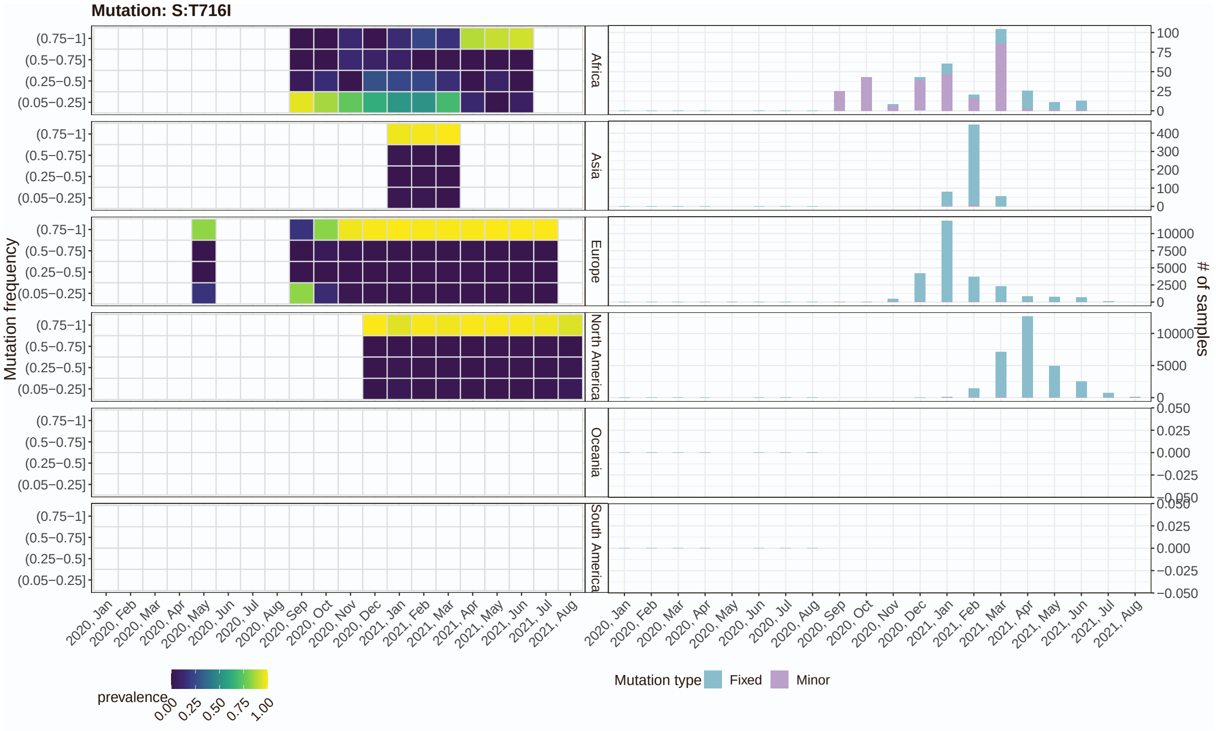

Figure S 21: **Mutant frequency and prevalence variation in time of mutation: S:T716I. Related to Figure 3.** Please refer to the caption of Figure S 3

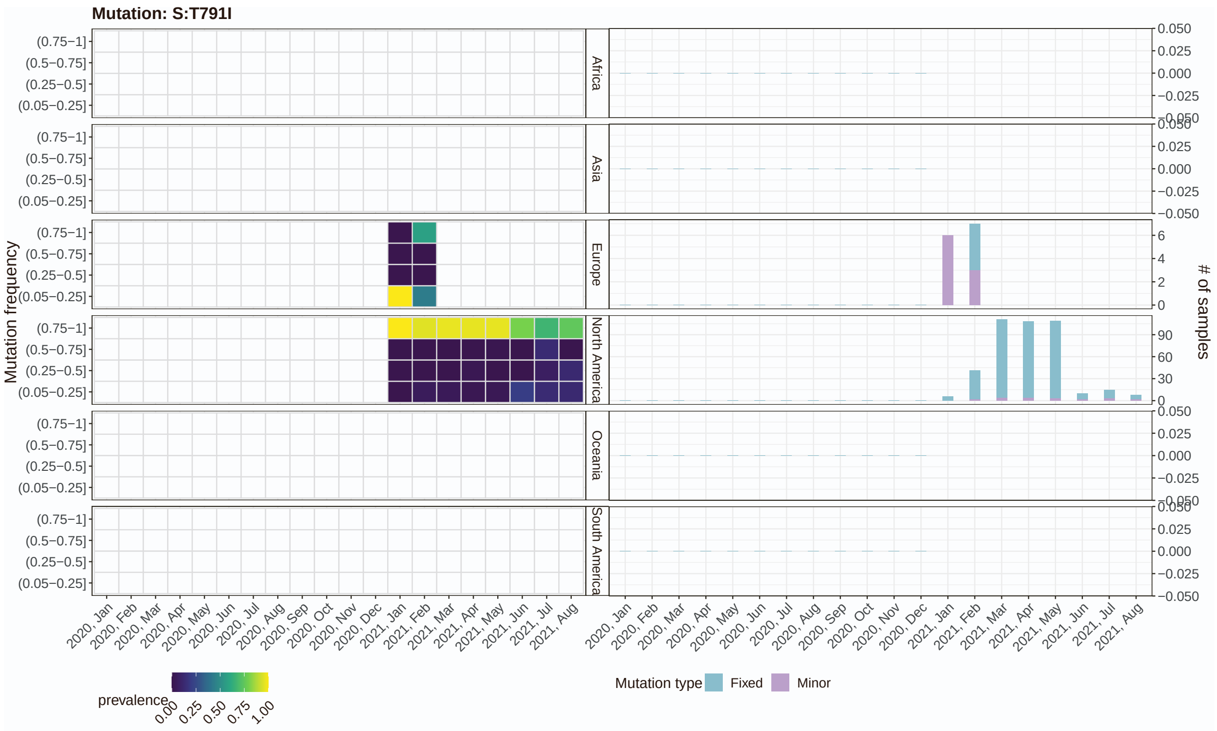

Figure S 22: **Mutant frequency and prevalence variation in time of mutation: S:T791I. Related to Figure 3.** Please refer to the caption of Figure S 3

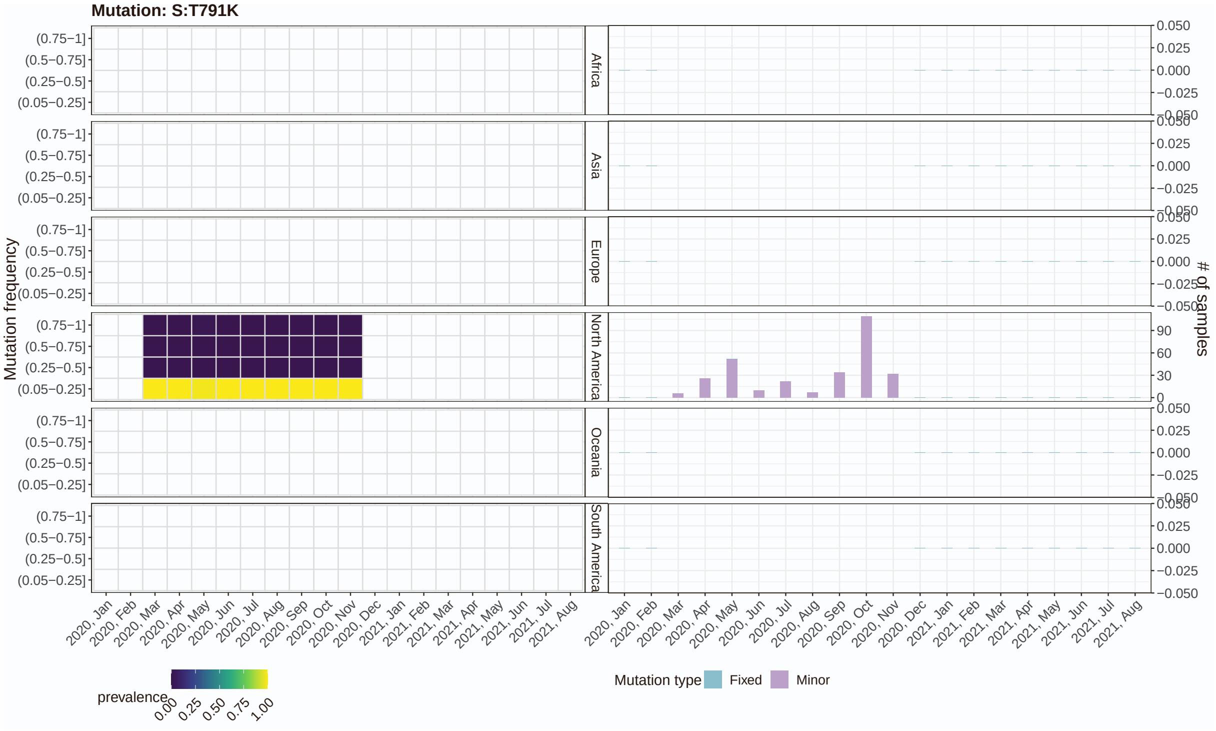

Figure S 23: **Mutant frequency and prevalence variation in time of mutation: S:T791K. Related to Figure 3.** Please refer to the caption of Figure S 3

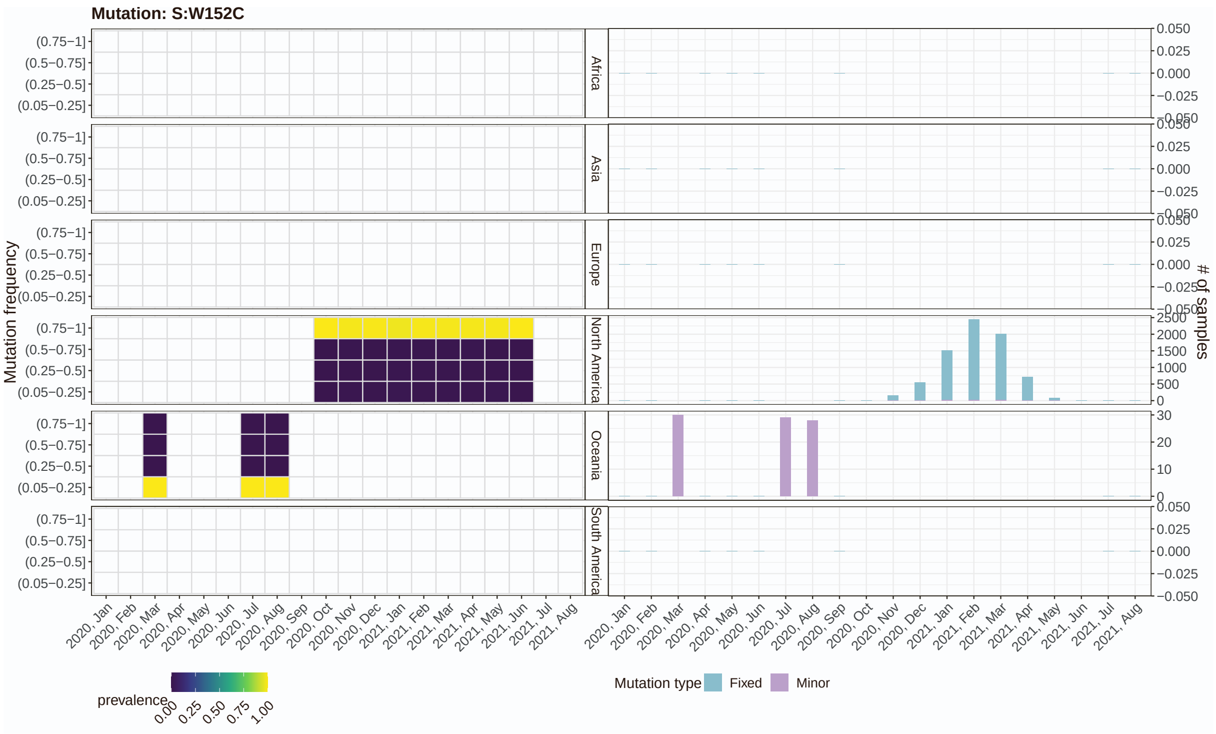

Figure S 24: **Mutant frequency and prevalence variation in time of mutation: S:W152C. Related to Figure 3.** Please refer to the caption of Figure S 3

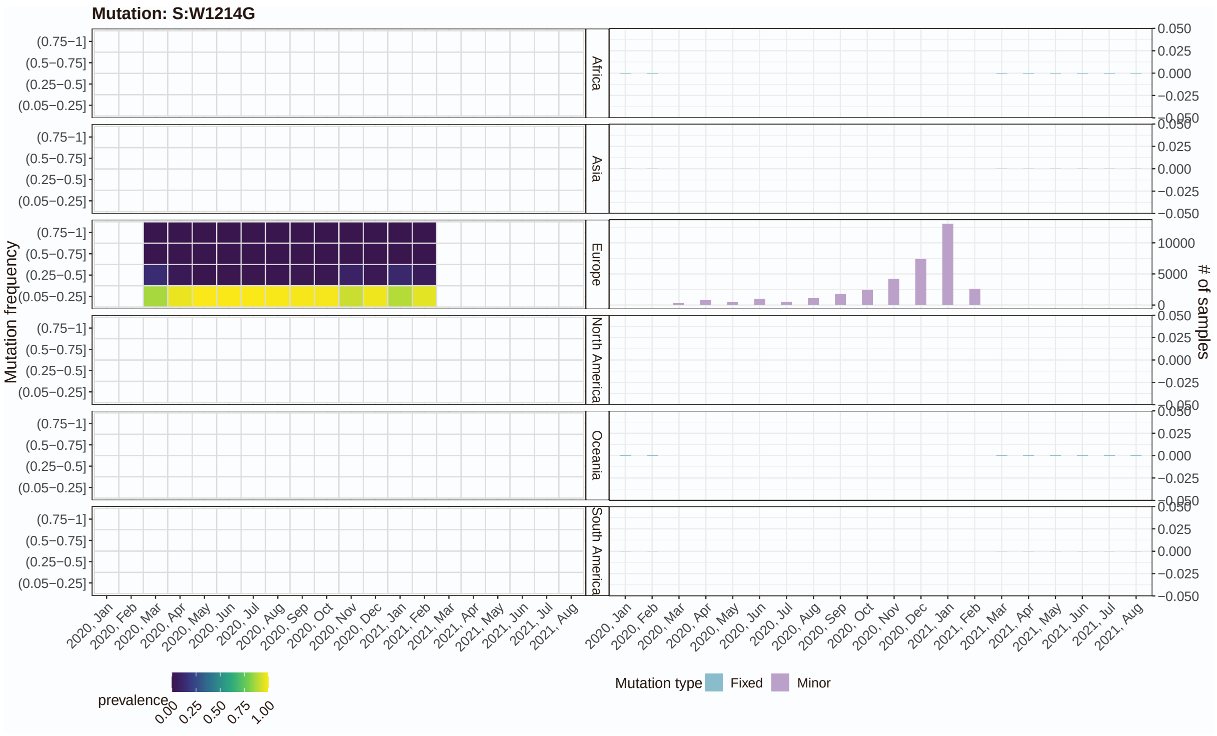

Figure S 25: **Mutant frequency and prevalence variation in time of mutation: S:W1214G. Related to Figure 3.** Please refer to the caption of Figure S 3

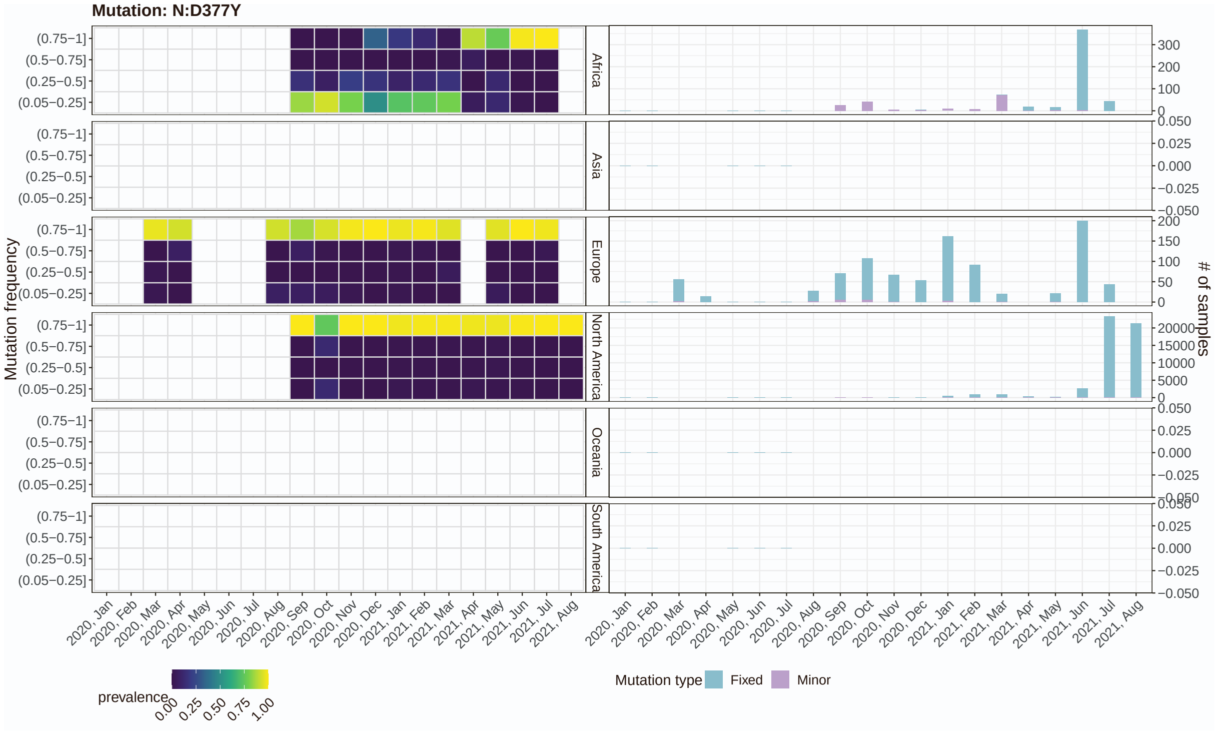

Figure S 26: **Mutant frequency and prevalence variation in time of mutation: N:D377Y. Related to Figure 3.** Please refer to the caption of Figure S 3

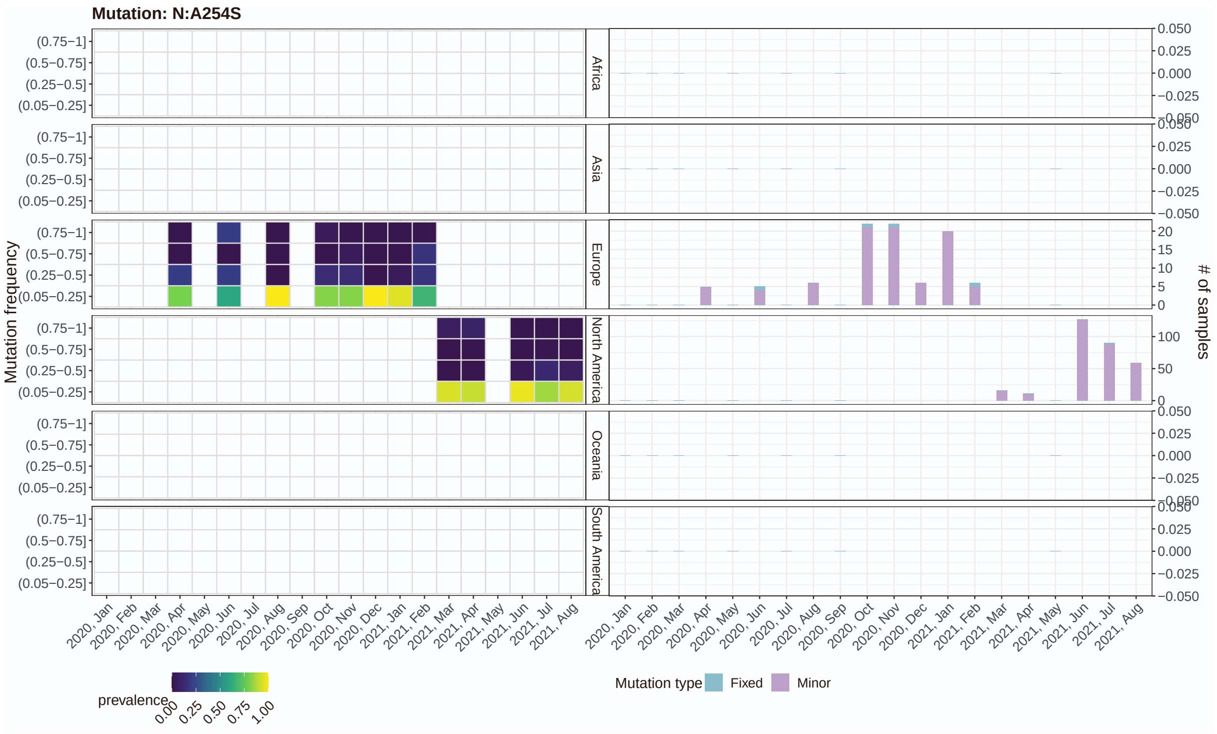

Figure S 27: **Mutant frequency and prevalence variation in time of mutation: N:A254S. Related to Figure 3.** Please refer to the caption of Figure S 3

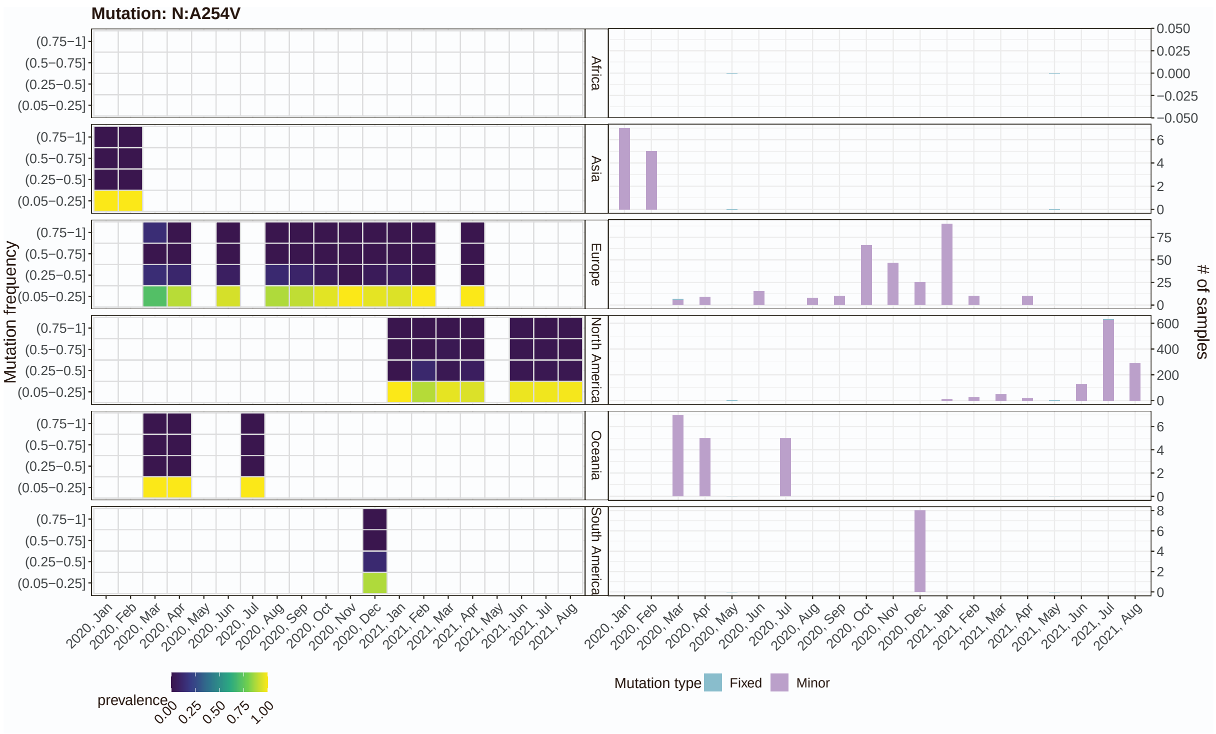

Figure S 28: **Mutant frequency and prevalence variation in time of mutation: N:A254V. Related to Figure 3.** Please refer to the caption of Figure S 3

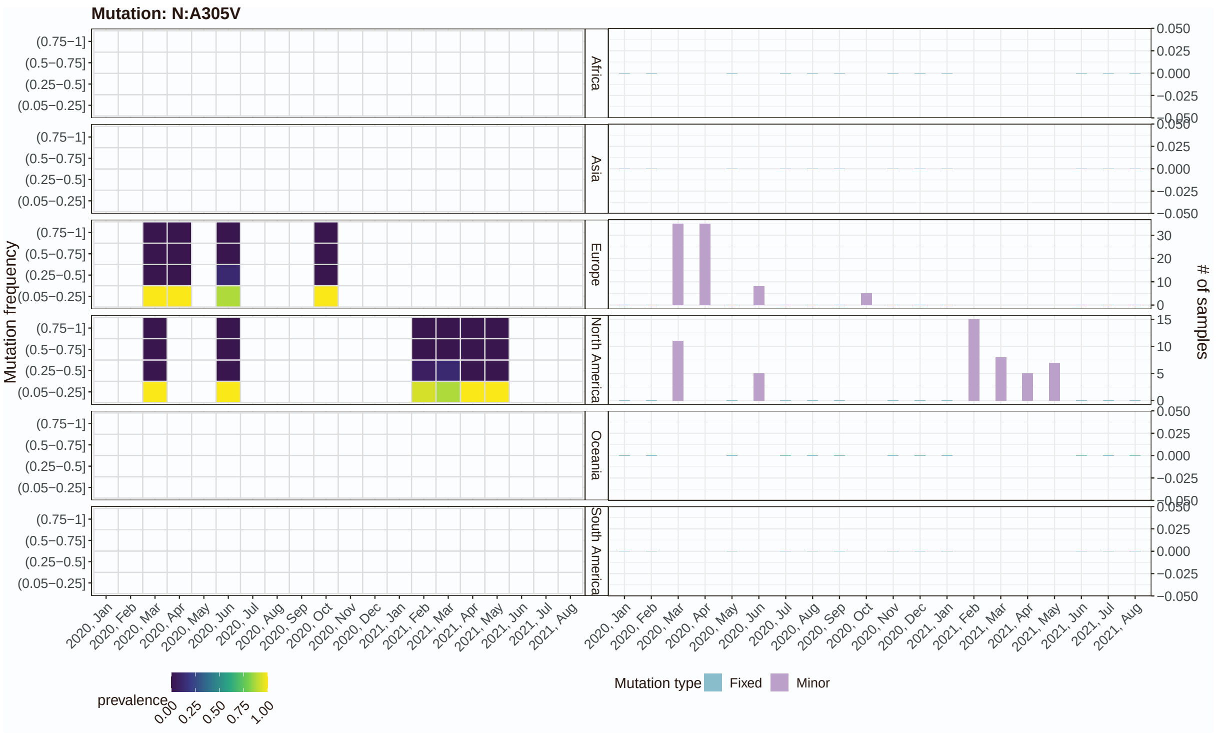

Figure S 29: **Mutant frequency and prevalence variation in time of mutation: N:A305V. Related to Figure 3.** Please refer to the caption of Figure S 3

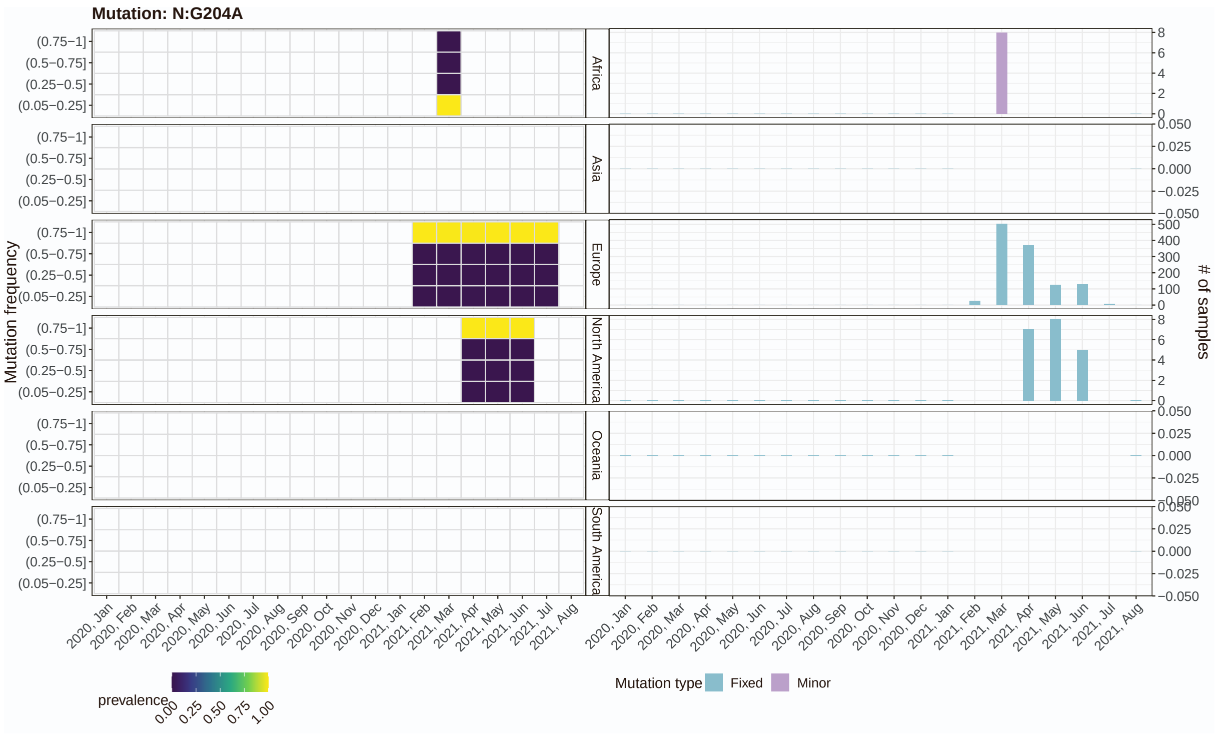

Figure S 30: **Mutant frequency and prevalence variation in time of mutation: N:G204A. Related to Figure 3.** Please refer to the caption of Figure S 3

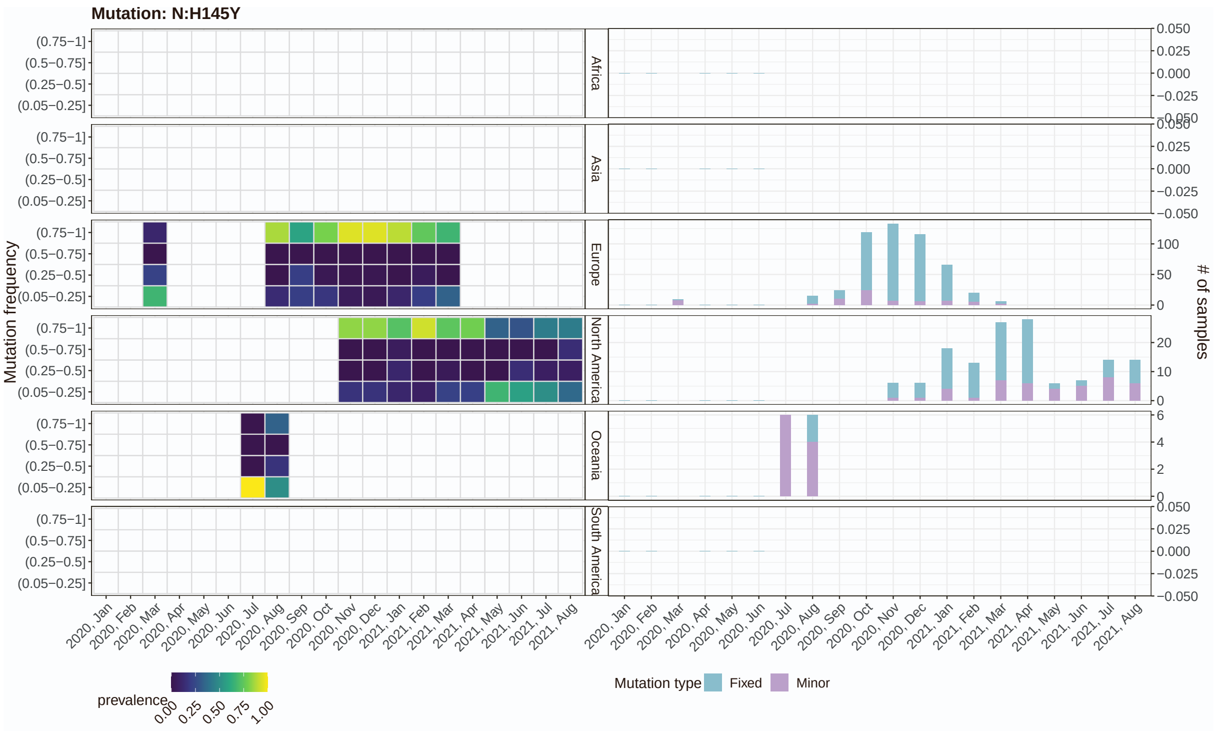

Figure S 31: **Mutant frequency and prevalence variation in time of mutation: N:H145Y. Related to Figure 3.** Please refer to the caption of Figure S 3

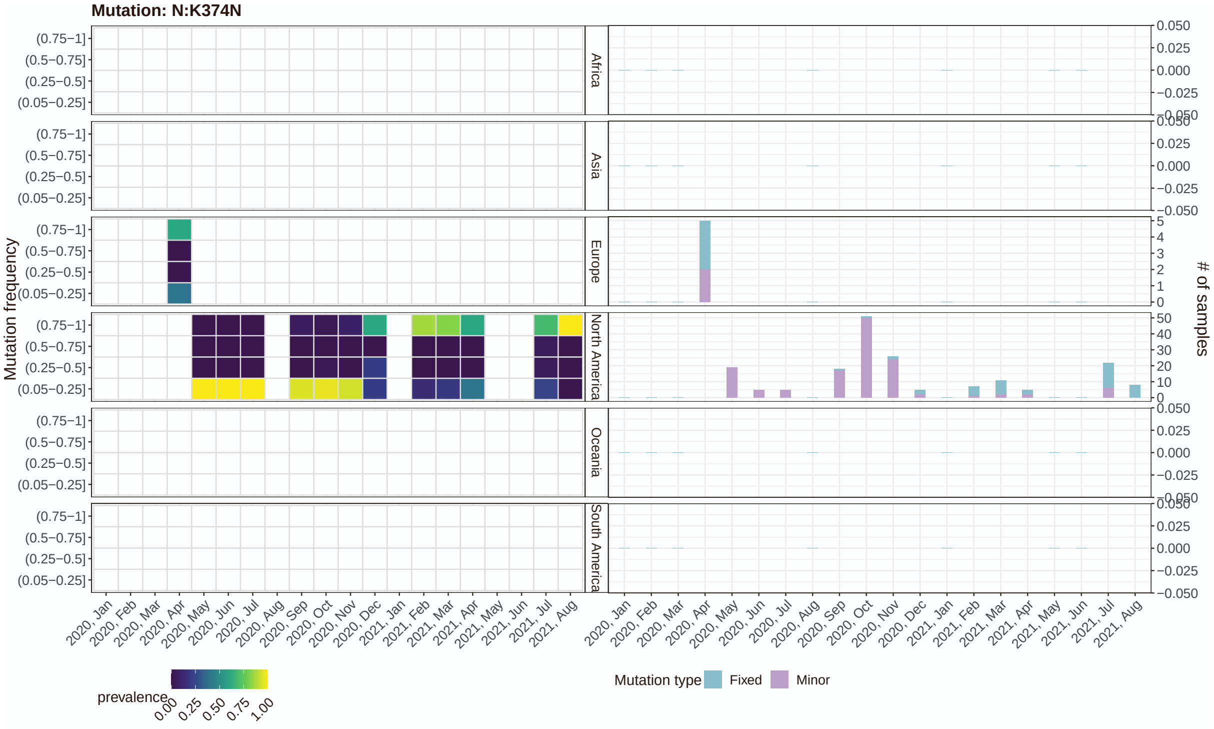

Figure S 32: **Mutant frequency and prevalence variation in time of mutation: N:K374N. Related to Figure 3.** Please refer to the caption of Figure S 3

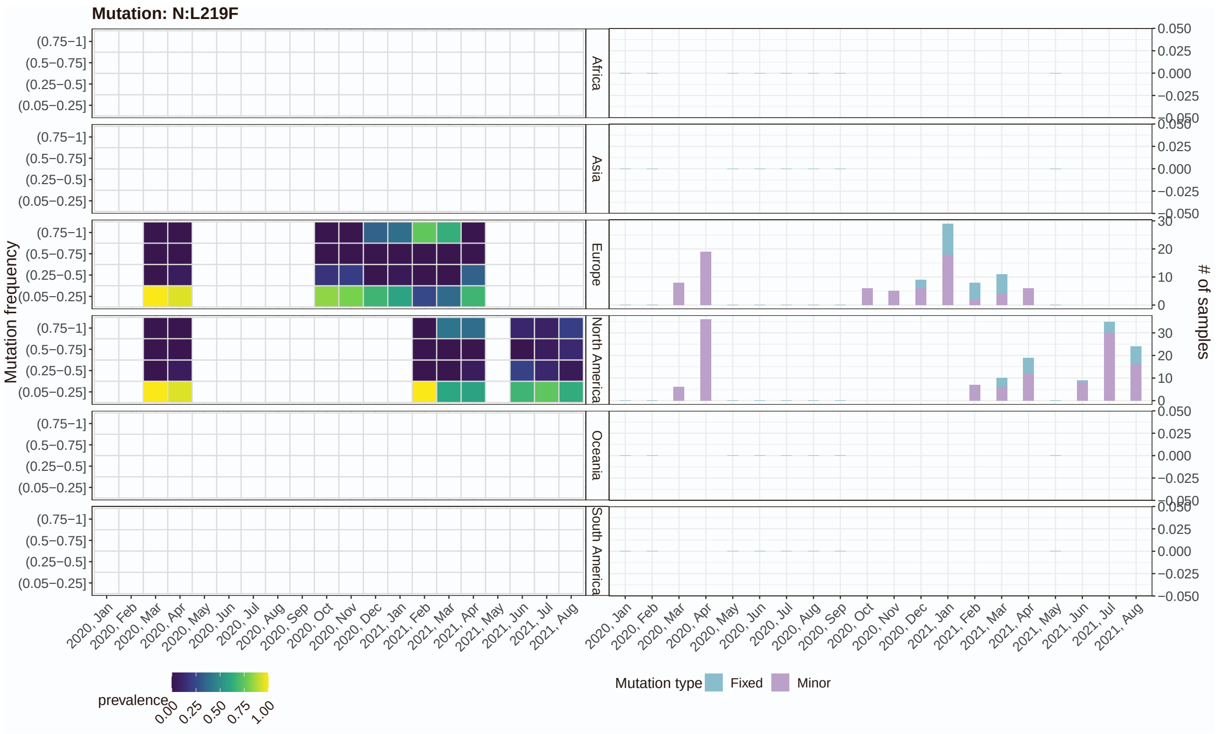

Figure S 33: **Mutant frequency and prevalence variation in time of mutation: N:L219F. Related to Figure 3.** Please refer to the caption of Figure S 3

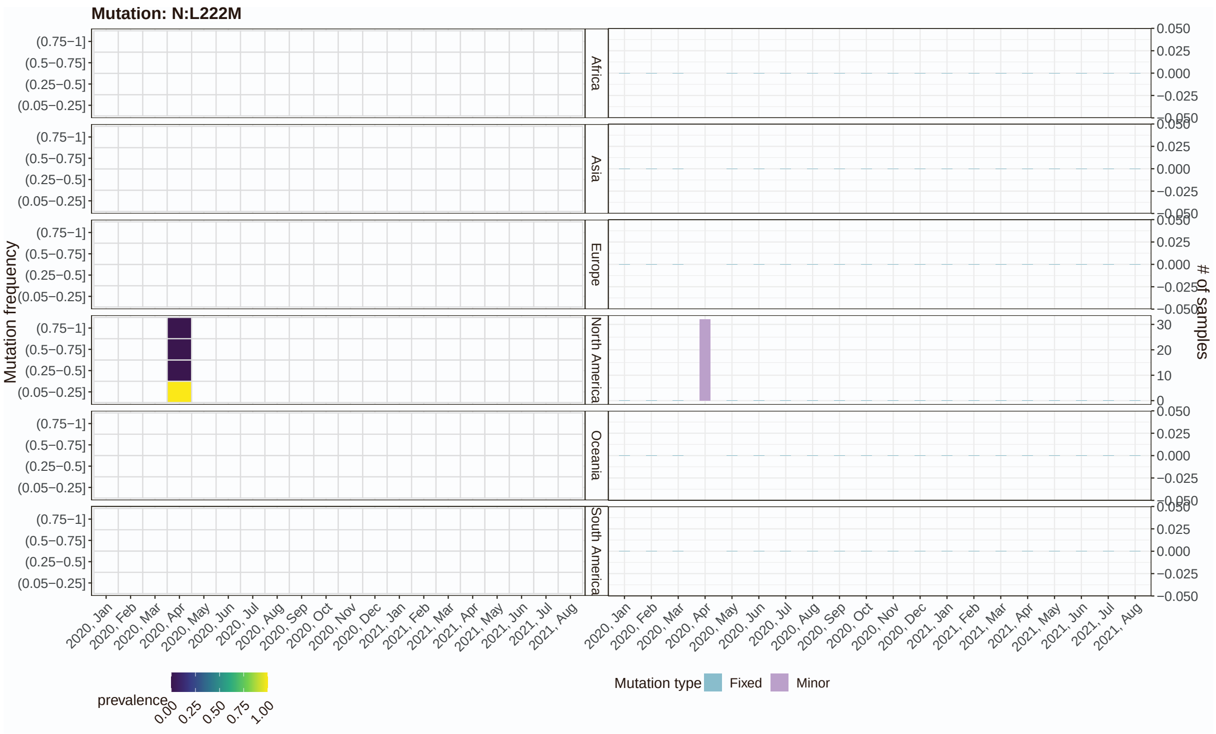

Figure S 34: **Mutant frequency and prevalence variation in time of mutation: N:L222M. Related to Figure 3.** Please refer to the caption of Figure S 3

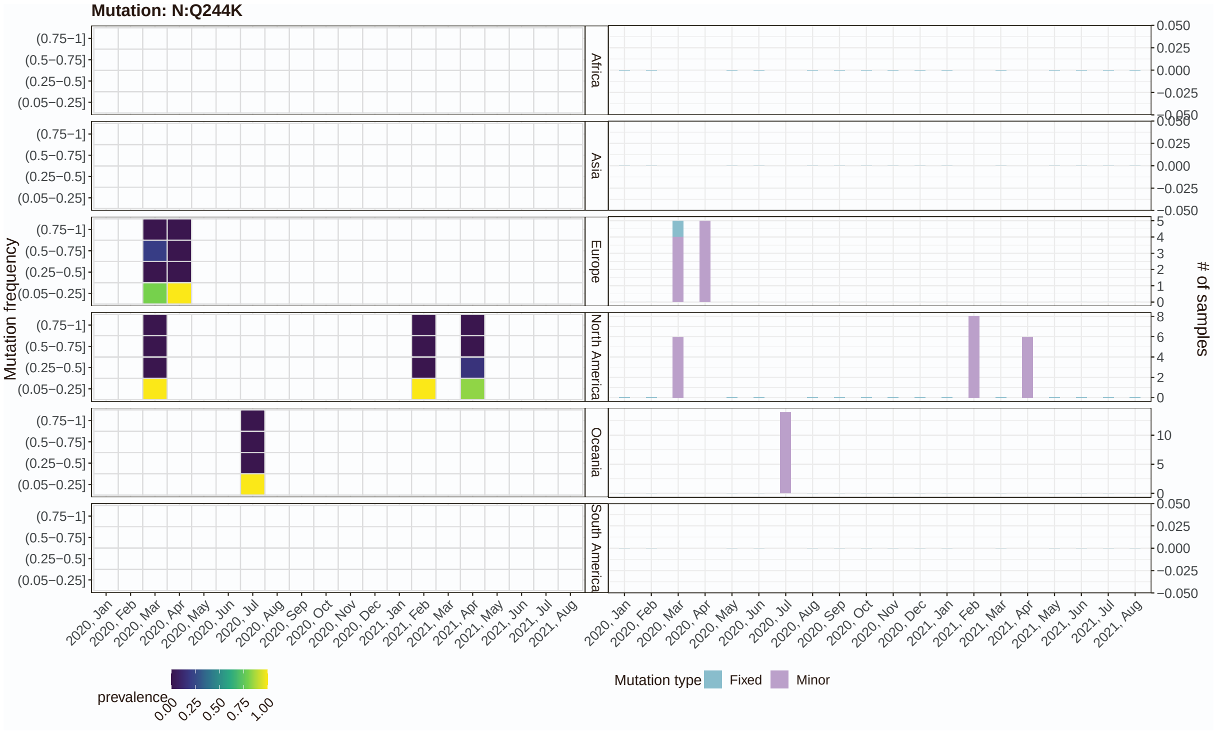

Figure S 35: **Mutant frequency and prevalence variation in time of mutation: N:Q244K. Related to Figure 3.** Please refer to the caption of Figure S 3

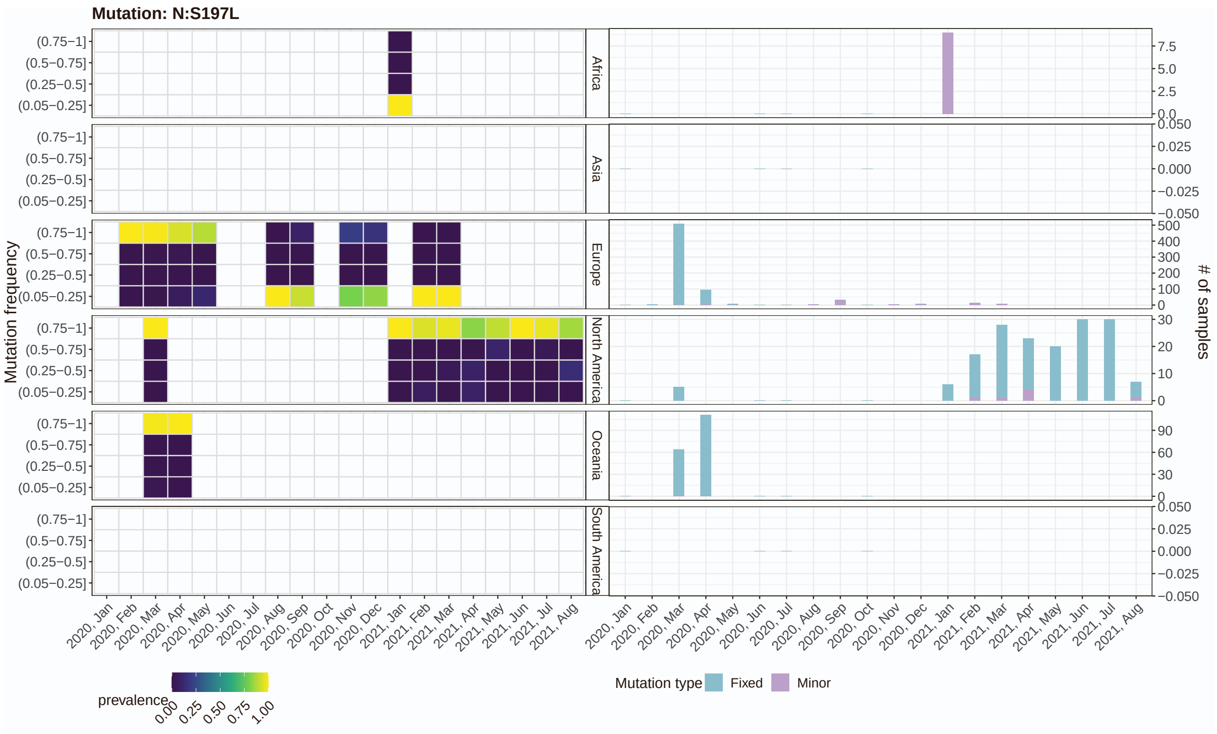

Figure S 36: **Mutant frequency and prevalence variation in time of mutation: N:S197L. Related to Figure 3.** Please refer to the caption of Figure S 3
